# The influence of EEG oscillations, heart rate variability changes, and personality on self-pain and empathy for pain under placebo analgesia

**DOI:** 10.1038/s41598-022-10071-9

**Published:** 2022-04-11

**Authors:** Vilfredo De Pascalis, Arianna Vecchio

**Affiliations:** Department of Psychology, Sapienza Foundation, Sapienza University of Rome, Via dei Marsi, 78, 00185 Rome, Italy

**Keywords:** Neuroscience, Physiology, Psychology, Health care

## Abstract

We induced placebo analgesia (PA), a phenomenon explicitly attenuating the self-pain feeling, to assess whether this resulted in reduced empathy pain when witnessing a confederate undergoing such pain experience. We recorded EEG and electrocardiogram during a painful Control and PA treatment in healthy adults who rated their experienced pain and empathy for pain. We derived HRV changes and, using wavelet analysis of non-phase-locked event-related EEG oscillations, EEG spectral power differences for self-pain and other-pain conditions. First-hand PA reduced self-pain and self-unpleasantness, whereas we observed only a slight decrease in other unpleasantness. We derived linear combinations of HRV and EEG band power changes significantly associated with self-pain and empathy for pain changes using PCAs. Lower Behavioral Inhibition System scores predicted self-pain reduction through the mediating effect of a relative HR-slowing and a decreased midline ϑ-band (4–8 Hz) power factor moderated by lower Fight-Flight-Freeze System trait scores. In the other-pain condition, we detected a direct positive influence of Total Empathic Ability on the other-pain decline with a mediating role of the midline β2-band (22–30 Hz) power reduction. These findings suggest that PA modulation of first-hand versus other pain relies on functionally different physiological processes involving different personality traits.

## Introduction

Empathy for pain is a complex phenomenon that allows the observer to understand and share other-pain sensory and emotional qualities. Research has shown that the nervous system of people experiencing another person's pain may react as if they felt that pain themselves^1,2^.

Among EEG/MEG and transcranial magnetic stimulation (TMS) studies, a frequently confirmed finding is that observing noxious, compared with neutral body events, produces the so-called suppressions of mu (7–12 Hz) and β (13–30 Hz) oscillations^3–6^, known to reflect the sensorimotor activity. Avenanti and colleagues^7–9^, using TMS, found smaller motor evoked potentials when participants attended video clips displaying needle injections than seeing touch at the exact location, suggesting motor inhibition in the sensorimotor cortex. In contrast, in a later study, Riečanský and colleagues^10^ found increased motor readiness and activation in the sensorimotor cortex, as expressed by increased central β (13–30 Hz) and mu (7–12 Hz) desynchronization when participants saw videos depicting painful needle injections than nonpainful control conditions. Indeed, they later observed that the activation of the sensorimotor cortex became more robust with increasing illusory ownership of the observed hand^11^. More recently, Riečanský et al.^12^ suggested that the facilitation of movement they had observed with needle-in-hand reflects an increased readiness for a defensive motor reaction of active avoidance (fear) or escape behavior.

In sum, research focusing on the effects of empathy on information processing produced heterogeneous results. To demonstrate shared neural functions of first-hand pain and empathy for pain, we should first highlight shared neural activity to self and other-pain modulation induced by a placebo analgesia (PA) treatment. PA is the effect of pain reduction that follows the administration of an inert treatment recommended as a potent pain killer^13,14^.

Then we should demonstrate that the PA modulation of shared neural activity is causally linked to both self-pain and empathy for pain changes. In addition, the influence of individual pain sensitivity-related traits on self-pain and dispositional empathy traits on other-pain changes should also be evaluated (see^15^ for a detailed commentary).

The individual dispositional effect on PA responding can be studied using the more recent revision of J. A. Gray's original Reinforcement Sensitivity Theory (rRST)^16–18^. The rRST has postulated three major neurobiological systems controlling approach and avoidance behaviors: the behavioral approach system (BAS), the behavioral inhibition system (BIS), and the fight-flight-freeze system (FFFS). All forms of appetitive stimuli activate the BAS. The BIS is linked to anxiety and triggered by all forms of goal conflict, whose function is to inhibit ongoing behavior and scan the environment. The FFFS is the primary system responsible for fear (active avoidance). If a threat requires an attack, both BIS and the FFFS are activated for a fight^16^. Following this theoretical stream, the Reinforcement Sensitivity Theory-Personality Questionnaire (RST-PQ)^19^ has been proposed as an excellent tool to measure BAS, BIS, and FFFS, wherein the last two traits are conceptualized as two interacting systems with different functional properties and distinct neuropsychophysiological functioning.

Research has demonstrated the EEG ϑ (4–8 Hz) activity as linked to goal conflict^20^ and BIS^21^. Research on fear conditioning in humans has highlighted increased γ oscillations in the occipital and prefrontal regions, increased ϑ oscillations in the posterior and lateral-frontal areas, and decreased α and β oscillations in the parietal and occipital regions, with the presence of such oscillations in the somatosensory cortex and insula^22^. Lyby and colleagues^23^ demonstrated that individuals higher in dispositional pain-related fear had decreased PA responding in the subjective report and event-related potentials. In line with these findings, we have recently obtained smaller reductions in pain ratings and smaller decreases in the P2 and P3 amplitudes of the ERP elicited by electric stimuli in higher FFFS trait scorers^24^. However, links between neuroticism-related traits and placebo responses are less consistent^25,26^.

Rutgen and colleagues^27^, eliciting ERPs to painful electrical stimuli, found that PA reduces measures of self-pain, other-pain, and the P2 amplitude of the ERPs. Their findings demonstrated that the modulation of first-hand pain in an equivalent mode also modulates empathy for pain. In our recent attempt to replicate and extend Rutgen et al.^27^ findings, we obtained that PA treatment reduced self-pain together with P2 and P3 peak amplitudes but not empathy for pain, indicating that different neural processes govern empathy and direct pain experiences. Additionally, in a previous study of our own, we examined the influence of reward sensitivity (RST-PQ), heart rate (HR) dynamics, and EEG-delta activity on tonic pain reduction by a PA treatment^28^. We found that a linear compound of HR slowing and enhanced EEG delta activity to PA treatment explains a substantial portion of the variance in PA responding. We observed that the Reward-Interest facet of the BAS and Involuntariness in pain reduction positively mediated this link.

Based on the above-reported observations, the main aim of the present exploratory study was to extend our previous ERP^24,27^, and HR^28^ placebo findings to event-related EEG oscillation and HR variability (HRV) changes as induced by phasic painful stimulations. We aimed to highlight the mutual influence of HRV indexes and non-phase locked event-related oscillations (1–40 Hz) on self-pain and empathy for pain changes. In line with earlier literature findings^10–12^, we expected a link between changes in β and α activities induced by PA treatment and self-pain changes. We also expected to disclose significant associations of BIS and FFFS motivational personality traits, as measured by the RST-PQ, with self-pain and other-pain changes. Finally, using conditional process analyses, we expected to evaluate the direct influence of BIS (passive avoidance) on self-pain relief following PA treatment and the role of FFFS trait and physiological indexes in this relationship. We predicted that people with lower BIS and FFFS scores should display higher reductions of self-pain and empathy for other pain, and vice versa, for higher levels of these traits. Finally, we also included measures of dispositional empathy facets, as obtained by the Empathy Component Questionnaire^29^ (ECQ), to test the influence of empathy as a trait on other-pain changes and highlight the consequent indirect effect of physiological factors in this relationship. We expected that higher levels of empathy trait should be associated with higher other-pain reductions induced by the PA treatment.

Finally, we demanded to see if our previous PA findings obtained for tonic pain^28^ are valid for phasic pain. We expected higher HR slowing and slow event-related EEG oscillations to be associated with PA-induced pain reduction.

## Methods

### Participants

Participants were 63 neurotypical right-handed university student volunteers, aged between 18 and 29 years (32 women: M = 21.56, SD = 2.41, men: M = 23.03, SD = 2.63). We excluded one male participant from data analyses because we detected outliers in his data. Thus, only data from 62 participants were analyzed.

The experimental protocol was conducted under the Helsinki Declaration (1964) and approved by the Institutional Review Board (IRB) of the Department of Psychology of Sapienza University of Rome (protocol number 0001291 issued on 07/12/2017). Informed consent was obtained from each participant (see Supplement S1 for more details).

The study is powered enough to detect a medium-sized effect (*f* = 0.40) in multiple regression, with two-tailed α = 0.05, power = 0.80, which requires N = 52.

### Questionnaires

The participants completed the RST-PQ^19^ measuring three major systems: the BAS, BIS, FFFS. The BAS is composed of the following facets: *Goal-Drive Persistence* (BAS-GDP), *Reward Interest* (BAS-RI), *Reward Reactivity* (BAS-RR), and *Impulsivity* (BAS-I). The total BAS (BAS-TOT) measure is obtained by summing the BAS-GDP, BAS-RI, BAS-RR, and BAS-I scores.

We also administered the ECQ^29^ consisting of five facets. From The ECQ facets, we derived the following principal scores (see^29^): *Cognitive Empathy* (CE), *Affective Empathy* (AE), *Empathic Drive* (ED), *Total Empathic Ability* (TEA), and *Cumulative Total Empathy* (CTE) scores. More details are provided in Supplement S1. Participants also completed the state anxiety form of the State-Trait Anxiety Inventory (STAI-Y1)^30^.

### Experimental trials and treatments

**Figure 1:**
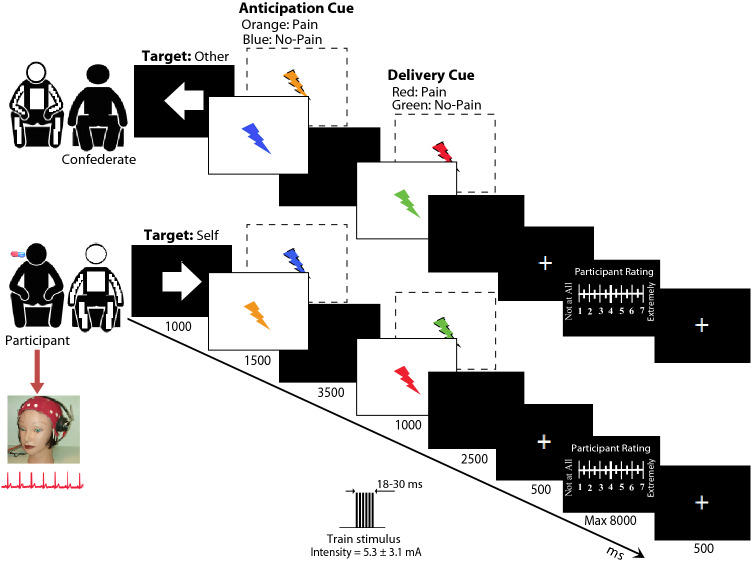
Structure and timeline for self-pain and other pain trials. An arrow cue (1000 ms) indicated the target of the upcoming electric stimulation (self, other). The arrow was followed by an anticipation cue (1500 ms) indicating the intensity of the upcoming electric stimulation (blue flash indicates a nonpainful stimulus, orange flash indicates a painful one). After a waiting interval of 3500 ms, a delivery cue (1000 ms) was presented concurrently with stimulus train delivery (duration from 18 to 30 ms): red flash represents a painful stimulus, green flash represents a nonpainful one. After 3000 ms, pain and unpleasantness ratings were collected (max 8000 ms) in about one-third of all trials.

To investigate pain-related empathy, we benefit from a known paradigm developed by Singer and colleagues^31^. Rutgen et al.^27^ and ourselves^24^ employed this paradigm to test empathic experience wherein the object of empathy experience was a real person seated on the left side of the participant chair (see Fig. 1). In the self-pain condition, participants were exposed to individually calibrated, short-lasting painful electric stimuli (duration from 18 to 30 ms) and nonpainful electric stimuli delivered to the back of their right hand. In the other-pain condition, participants experienced empathy for the pain of the confederate seated next to whom we delivered painful stimuli and nonpainful electric stimuli to the back of her right hand. A Digitimer DS5 Isolated Bipolar Constant Current Stimulator (Digitimer Clinical and Biomedical Research Instruments) generated electrical stimulations. We used a planar concentric surface wasp point electrode (7 mm diameter) with a central platinum pin (WASP electrode, Specialty Developments, Germany). Planar concentric electrodes stimulate the superficial skin layer^32^ involving the A-δ fibers and A–C terminals^33^, making them reliable in producing pain-evoked potentials like that obtained with laser stimulations^34^. The inclusion of nonpainful stimuli in the present study only tested the expected effects that placebo analgesia should only affect ratings of painful stimuli. Each self-pain and other-pain condition took ~ 16 min, wherein 36 painful stimuli and 36 nonpainful stimuli were delivered respectively to the participant and the confederate in random order.

We used the e-prime 2.0 system to program the trial structure of the empathy for pain task. We benefited from a trial structure like that used by Rutgen et al.^27^, with a timeline adapted to make possible an HRV analysis of the ECG activity. A 19" color LCD monitor (1400 × 900 resolution and 75 Hz vertical refresh rate) presented visual stimuli, with the participants seated at a distance of 80 cm. Horizontal and vertical visual angles of all visual stimuli were 5.2° and 6.9°, with a mean luminance of 22.5 cd/m^2^. The trial structure, stimulation, and timing are provided in Fig. 1 and Supplement S2. In the self-pain condition, participants rated, after the presentation of a painful stimulus, their experienced pain and unpleasantness on a numerical 7-point Likert scale from 1 ("barely perceptible but no painful") to 7 ("unbearable pain") to obtain a numerical pain score (S_NPS). A similar numerical scale served to rate the unpleasantness score (S_NUS). Equivalently, participants used similar 7-point Likert scales to rate the inferred pain and unpleasantness experienced by the confederate (O_NPS and O_NUS, respectively, for numerical pain and unpleasantness of the other). Pain and unpleasantness ratings were presented in a quasi-random order. We subtracted S_NPSs, S_NUSs, and O_NPSs, O_NUSs rated during Placebo from the corresponding scores rated during Control treatment to obtain numerical pain and unpleasantness difference scores S_NPDSs, S_NUDSs, and O_NPDSs, O_NUDSs, respectively. These difference scores served as bases for statistical analyses.

### Procedure

This experiment consisted of two sessions conducted over two days. Participants first signed approved informed consent forms during the first session and then completed the RST-PQ and EPQ. The participant and the confederate were invited for electrophysiological recordings on the second experimental day. The confederate was always a female, as well as the experimenter. Before EEG recordings, each participant underwent a psychophysical pain calibration procedure using the method of limits to determine a reliable electrical stimulation intensity for painful and no painful stimuli. Electric stimulations were delivered through a Digitimer DS5A stimulator via an e-prime 2.0 program administering trains of electrical stimuli with increasing current intensity. Pain thresholds were determined using a train of 6 pulses (duration of one pulse = 2 ms; pause = 2 ms; train duration = 24 ms) delivered by planar concentric electrodes on the back of the right hand.

The first electrical pulse train was 0.5 mA, and then intensity was progressively increased using an ascending method of limits with current increments randomly ranging between 0.25 to 0.5 mA until the subjective threshold pain was reached. The pain threshold procedure stopped when the average pain, perceived in 3 consecutive electric trains, surpassed the threshold at a critical rating level of 6 ("extremely painful, but bearable"). The electric shocks were then presented in a descending intensity sequence, and the participant was asked to indicate when the stimulus was no longer painful and when he/she did feel the pulse as perceptible but not painful. This procedure was repeated two times. Only averaged data from the final two series were used for the threshold calculations. The interval between the end of an evaluation and the beginning of the next was between 5 and 7 s. After each stimulation, the participant expressed a value on a 7-point Likert scale, ranging from 1 ("barely perceptible but no painful") to 7 ("unbearable pain"). We selected as painful stimuli those rated with a value of 6, and as nonpainful stimuli, those rated as 2 ("well perceptible but no painful"). The duration of the threshold assessment was approximately 7 min. The mean (M) and standard deviation (SD) of current intensity obtained for pain stimuli across all participants were: M = 5.3 mA, SD = 3.1 mA. The maximum current intensity ranged from 4 to 10 mA. It never exceeded the intensity value of 10 mA due to the limit required by our institutional ethics committee in the whole experimentation procedure.

After the calibration procedure, participants were exposed to two experimental pain treatments: a Control and a PA treatment. The Control treatment lasted about 32 min: 16 min for self and 16 min for other-pain trials, with participants experiencing pain without any prescription. In the PA treatment, each participant had to ingest a placebo capsule and then participate in a pain manipulation procedure known to reduce the first-hand experience of pain. The PA treatment made it possible to test whether it modulates empathy for pain. Control and PA treatments turned up in a counterbalanced order across participants. The pain manipulation procedure (10 min) was always preceding the experimental task in the PA treatment. Pain manipulation aims to engender strong expectations in the participant for lower pain in response to painful stimulation. The placebo capsule was recognized as an expensive and highly effective "pain killer" in the PA treatment.

The experimenter gave each participant the following suggestion: "I ask you to ingest a capsule containing an experimental highly effective and short-lasting pain reliever substance. We are testing two different dosages. One dosage produces a powerful analgesic, while the other should produce a minor analgesic effect. For practical design reasons, neither the experimenter nor the participants know which dosage we are now using. We can guarantee that the administering capsule is a highly effective pain killer with rapid onset and short duration free from side effects or contraindications as certified by doctors of our university's medical center. The action of the drug will reach the maximum effectiveness within 15–20 min, and its analgesic effect will be fully lost after 60 min from the ingestion". We used this experimental protocol to avoid the "analgesic capsule" being a sham treatment since the participants were students in psychology courses. In this way, we tried to avoid any surprise effect on the EEG responses^35^. Although in this way, we may have reduced the placebo effect, we avoided biased effects caused by possible suspicions that in the capsule, there could be a sham drug^36^. Twenty minutes after ingesting the capsule, each participant was subjected to a pain-manipulation procedure^37^. The participant was delivered a series of 4 train pulses he/she rated medium intensity (i.e., 3 or 4) in the previous calibration phase, but we forced participants to believe that they were delivered stimuli of the same intensity they had previously rated 6. After administering each conditioning train, participants rated medication effectiveness in pain reduction by asking: "How effective is this medication in reducing your pain?".

The confederate did not receive any medication, and all participants were purposely informed about this. When PA was the first Treatment, it lasted at least 72 min in total: 20 min waiting to ensure drug absorption and action, 10 min for manipulation, 32 min for both self and other pain trials, and finally, 10 min relaxation waiting for assuring the offset of the analgesic drug's effect. At the end of the PA trials, participants had to relax for at least 10 min to guarantee the offset of the analgesic effect. The experimenter did not start the subsequent Control treatment until participants had confirmed the feeling of the termination of any analgesic effect on them. The partner was seated next to the participant's left side with the mandatory request to fix their gaze to the ground to prevent direct observation of the other. In addition, each participant also received a mandatory injunction to maintain a fixed eye on the screen and avoid directing the gaze to the confederate. We also informed both partners that their posture was video monitored and that the experimenter would stop the experiment if they looked at each other. During each experimental condition, painful stimuli were delivered to the participant with the previously calibrated intensity of 6. The testing session in total took about 1.9 h. At the end of the experiment, we dismissed participants after filling the state anxiety inventory (STAI-Y1).

### EEG recordings and wavelet analysis

EEG activity was recorded from 30 scalp sites according to the extended 10–20 system, with the addition of two earlobes electrodes (A1, A2) using 32-tin electrodes stretch Lycra cap with a ground electrode mounted between FPz and Fz (Electro-Caps, Eaton, OH, USA). The NuAmp acquisition system (Neuroscan Acquire 4.3, Compumedics Neuroscan Inc, Charlotte, North Carolina 28269, USA) with an online notch filter at 50 Hz. The reference electrode was at the linked earlobes [(A1 + A2)/2]. The electrode impedance was kept less than 5 kΩ. The EEG was recorded in DC mode (sampling frequency = 1000 Hz, gain = 200, bandpass = 0.01–100 Hz: Butterworth zero-phase filter with 24 dB/octave roll-off) with an online 50 Hz notch filter. Both vertical and horizontal eye movements and eye blinks were monitored. Trials contaminated by eye blinks, eye movements, or electromyographic (EMG) activity exceeding ± 75 μV at any electrode were excluded from the analyses. Then, the EEG signals were downsampled to 250 Hz and transformed to standard average reference to obtain reference-free recordings. We removed horizontal and vertical EOGs and EMG artifacts by extracting 1 to 3 out of 30 independent components (IC; using Infomax algorithm, Brain Products; Vision Analyzer 2.2.2, Gilching, Germany)^38^. We reconstructed the EEG trace into discrete, single-trial 1000 ms artifact-free epochs (from 33 to 36) that were time-locked to the offset of painful electric-train stimulus delivered to the participant and to the onset of red-spark visual cue for the painful stimulus delivered to the confederate (see Fig. 1) with a 500-ms prestimulus baseline. For each Treatment, we first calculated ERPs in self-pain and other-pain conditions. We subtracted ERPs in each stimulus condition from the corresponding EEG epoch to remove the phase-locked EEG activity from the EEG data.

A time–frequency (TF) representation based on the continuous Morlet wavelet transform (CMWT) of every single EEG epoch (explored frequencies: 1–40 Hz, 1 Hz step) was used to identify non-phase-locked (stimulus-induced) power modulations of oscillatory activities (for details see Supplement S3). To enhance EEG changes time-locked (but not phase-locked) to stimulus onset, the CMWT was applied to each trial. The Resulting TF power maps were then averaged across trials for each subject and within each pain condition. These maps express the average oscillation power as a function of time and frequency.

**Figure 2:**
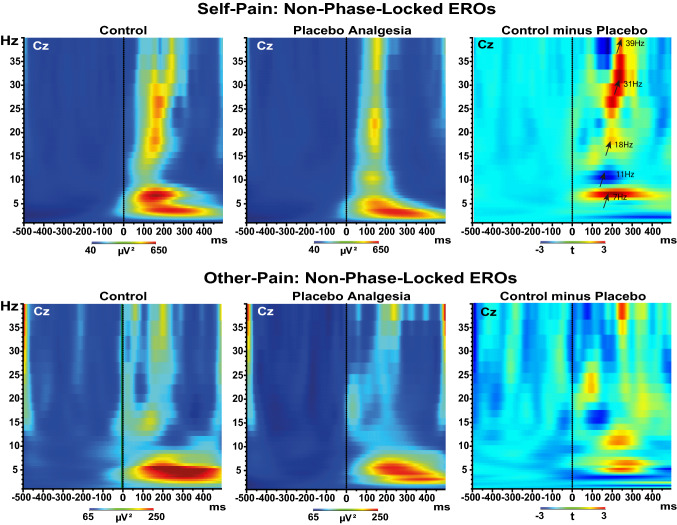
Grand averages across participants of single trials time–frequency (TF) estimation of non-phase locked (induced) oscillation power obtained by using the norm of the Morlet transform of EEG time-series recorded at Cz as elicited at the offset of painful electric train stimulus. Time is presented on the *x*-axis, and the vertical bar indicates stimulus offset at 0 ms. The frequency between 0 and 40 Hz is presented on the *y*-axis. Normed output spectral power values are coded on a color scale, the highest energy values appearing *red* and lower values *blue*. Data are baseline referenced, thus providing levels of positive power values relative to a reference period (from − 500 to − 50 ms). EEG changes for the Control and Placebo analgesia treatment during each self-pain (upper-panel) and other-pain (lower-panel) conditions. Right panels display the t-test differences between the two conditions. A power increase relative to baseline level can be observed in response to all stimuli during Control compared to Placebo treatments. This increase is pronounced in the self-pain between 100 and 250 ms. The maximum relative increases during the Control of TF power were at 7 Hz, 11 Hz, 18 Hz, 31 Hz, and 39 Hz, as shown by the arrows in the uppeR–Right panel. The power increases can be observed at all midline electrodes but are more assertive at central locations.

**Figure 3:**
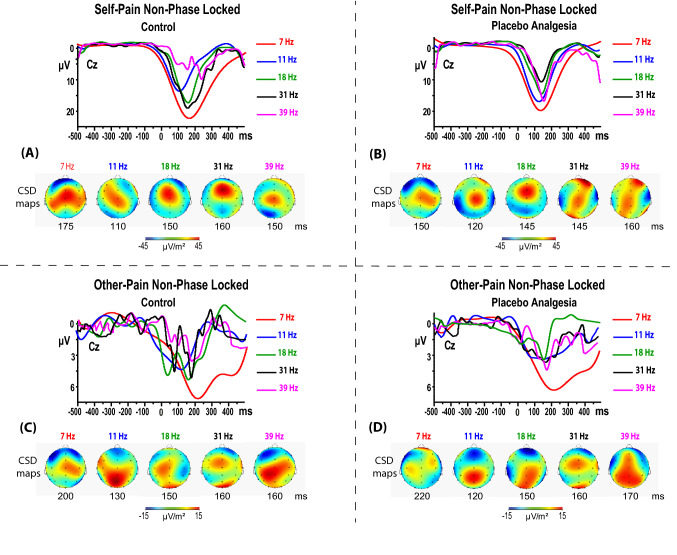
Wavelet-extracted **o**scillatory amplitude waveforms at frequency layers of 7, 11, 18, 31, and 39 Hz from the averaged wavelet-transformed single trials of the self-pain (upper-quadrant) and other-pain (lower-quadrant), respectively, for Control (**A**, **C**) and Placebo Analgesia (**B**, **D**) treatments (painful electric-train onset at time 0 ms). Color current source density maps (µV/m^2^) are reported at the bottom for each frequency of interest (7, 11, 18, 31, and 39 Hz) and the time corresponding to each maxima amplitude for each frequency.

We considered the mean TF real power of the prestimulus period (between − 500 and − 50 ms) as a baseline level. These baseline levels were subtracted from the prestimulus and post-stimulus power for each frequency step. Grand averages of induced TF representations of the power values at electrode Cz are displayed in Fig. 2 for first-hand pain and other conditions. We obtained significant t-values (see right side of Fig. 2) for the following five EEG dominant sub frequencies and time-intervals: ϑ (4–8 Hz, 50–250 ms); α (9–13 Hz, 100–200 ms); β_1_ (14–21 Hz, 100–200 ms), β_2_ (22–32 Hz, 100–180 ms), γ (33–40 Hz, 120–180 ms). We first obtained the maximum amplitude for each of these frequency bands of interest and the associated frequency (7, 12, 18, 31, and 39 Hz, respectively). We then computed the current source density (CSD, μV/m^2^) transforms of extracted wavelet waveforms at each frequency of interest mentioned above (for more details, see Supplement S3). We used the CSD transform as a spatial filter to identify the topographical source at maximum amplitude for each waveform of interest^39^. These CSD maps indicated that midline frontal (Fz), central (Cz), and parietal (Pz) are sensitive sites to experimental manipulations (Fig. 3).

### HR recordings

We recorded the electrocardiogram (ECG) using two beryllium copper electrodes (1.5 cm in diameter) with a sample rate of 100 Hz. We processed the continuous ECG recording signal with Kubios HRV Analysis 3.0.2 software^40^ to obtain the HRV measures used in the present study. Based on our previous HRV findings^28^, we selected the time domain, frequency domain, and sample entropy measures.

### Reduction of physiological variables

We derived Control minus Placebo difference scores (∆) in the R–R time interval that we labeled as ∆tHRV (ms), the standard deviation of normal-to-normal R–R interval (∆SDNN, ms), Low-Frequency power (∆LF power, 0.04–0.15 Hz), and High Frequency (∆HF power, 0.15–0.4 Hz), LF/HF ratio, Sample Entropy (∆S-Entr). More details on HR recordings and HRV are available in^40^ and Supplement S3.

For the EEG oscillation measures, to reduce skew, we derived Control minus Placebo difference scores of natural log transformation of TF mean power calculated for each of the ϑ, α, β_1_, β_2_, and γ frequency bands across Fz, Cz, and Pz leads.

We performed five varimax-rotated Principal Components Analyses (PCAs) to reduce data dimensionality, one for each of the five frequencies of interest and separately for self-pain and other-pain conditions, on the HR and EEG frequency indices (Supplement S4). Each of the five PCA involved six HRV difference indices, as reported above, and three EEG Control minus Placebo difference indices as obtained across Fz, Cz, and Pz midline scalp sites of interest. These analyses served to select (i) the EEG indices loading above the threshold of 0.40 in a factor together with HVR indices (j) to reduce problems of multicollinearity, for each EEG frequency of interest, in the subsequent analyses. Results of these preliminary analyses for self-pain and other-pain for ϑ, α, β_1_, β_2_, and γ EEG frequency bands of interest are reported in Table 1.

**Table 1:** Principal Component Analysis (PCA) factors as obtained using Pain minus Placebo differences scores of HRV and EEG frequency band power scores (N = 62).

Indices	Varimax rotated fact pattern														
	Self-Pain														
	ϑ (4–8 Hz)			α (9–13 Hz)			β1 (14–21 Hz)			β2 (22–32 Hz)			γ (33–40 Hz)		
	Fact1	Fact2	Fact3	Fact1	Fact2	Fact3	Fact1	Fact2	Fact3	Fact1	Fact2	Fact3	Fact1	Fact2	Fact3
∆tHRV (ms)	0.053	**− 0.404**	0.126	0.018	− 0.072	**− 0.673**	0.076	− 0.103	0.217	0.062	− 0.131	0.258	0.097	**− 0.763**	− 0.138
∆SDNN (ms)	0.055	− 0.041	**0.898**	0.027	**− 0.810**	− 0.320	0.022	− 0.044	**0.911**	0.031	− 0.015	**0.899**	0.054	− 0.114	**− 0.883**
LF power (nu)	**0.959**	− 0.028	0.042	**0.956**	− 0.047	− 0.069	**0.954**	0.027	0.069	**0.962**	0.056	0.064	**0.954**	− 0.005	− 0.040
∆HF power (nu)	**− 0.957**	0.024	− 0.058	**− 0.956**	0.061	0.029	**− 0.953**	0.002	− 0.091	**− 0.952**	0.097	− 0.077	**− 0.958**	− 0.041	0.061
∆LF/HF ratio	**0.972**	− 0.069	− 0.016	**0.973**	0.028	− 0.072	**0.980**	0.006	0.017	**0.976**	− 0.091	0.008	**0.978**	0.020	0.015
∆SamEn	− 0.005	0.094	**− 0.874**	0.001	**0.890**	0.015	0.022	− 0.043	**− 0.848**	0.018	0.001	**− 0.856**	0.007	− 0.074	**0.888**
∆ln (EEG Freq Pow: Fz)	0.039	**0.803**	0.021	− 0.013	0.108	0.227	0.038	**0.756**	0.109	− 0.019	**0.556**	0.046	− 0.165	− 0.324	0.160
∆ln (EEG Freq Pow: Cz)	0.096	**0.664**	− 0.096	0.131	**− 0.424**	**0.545**	− 0.197	**0.853**	− 0.147	− 0.092	**0.826**	− 0.149	0.143	**0.883**	− 0.001
∆ln (EEG Freq Pow: Pz)	− 0.192	**0.643**	0.081	− 0.187	0.073	**0.728**	0.199	**0.799**	− 0.220	0.012	**0.834**	− 0.166	− 0.057	**0.398**	0.015

Indices	Other-Pain														
	ϑ (4–8 Hz)			α (9–13 Hz)			β1 (14–21 Hz)			β2 (22–32 Hz)			γ (33–40 Hz)		
	Fact1	Fact2	Fact3	Fact1	Fact2	Fact3	Fact1	Fact2	Fact3	Fact1	Fact2	Fact3	Fact1	Fact2	Fact3
∆tHRV (ms)	− 0.027	0.021	**0.853**	− 0.043	**0.794**	− 0.187	− 0.021	− 0.076	**0.849**	− 0.052	− 0.031	**0.856**	− 0.005	**0.843**	− 0.184
∆SDNN (ms)	**− 0.610**	− 0.172	− 0.290	**− 0.534**	**− 0.415**	− 0.225	− 0.602	− 0.175	− 0.287	**− 0.605**	− 0.307	**− 0.410**	**− 0.637**	− 0.267	0.104
LF power (nu)	**0.954**	− 0.015	0.038	0.945	0.111	− 0.013	**0.952**	0.005	0.047	**0.950**	− 0.124	0.047	**0.940**	0.029	− 0.016
∆HF power (nu)	**− 0.948**	0.059	− 0.079	− 0.925	− 0.156	− 0.117	**− 0.940**	− 0.071	− 0.081	**− 0.938**	0.095	− 0.087	**− 0.944**	− 0.054	− 0.035
∆LF/HF ratio	**0.948**	− 0.043	0.008	**0.945**	0.070	0.032	**0.945**	0.072	0.012	**0.949**	− 0.081	0.003	**0.929**	0.014	0.089
∆SampEn	0.266	0.043	**0.783**	0.188	**0.823**	0.121	0.270	− 0.030	**0.796**	0.234	− 0.255	**0.769**	0.316	**0.731**	0.005
∆ln (EEG Band Pow: Fz)	0.023	**0.755**	− 0.252	− 0.125	0.151	**0.811**	0.236	**0.579**	− 0.144	0.128	**0.783**	− 0.070	− 0.231	0.332	**0.504**
∆ln (EEG Band Pow: Cz)	− 0.040	**0.679**	0.109	0.146	− 0.210	**0.755**	0.038	**0.798**	− 0.139	− 0.054	**0.677**	− 0.066	0.274	− 0.205	**0.733**
∆ln (EEG Band Pow: Pz)	0.038	**0.680**	0.149	**− 0.485**	0.157	0.235	− 0.045	**0.753**	0.175	− 0.211	**0.699**	− 0.074	− 0.052	− 0.127	**0.712**

Significant values are in bold.

We got three factors solution (Fact1, Fact2, and Fact3) by separate PCA analyses including HRV indices and, singly, each EEG band power of ϑ, α, β1, β2, and γ rhythms, performed respectively for Self- and Other-Pain conditions.

Pain minus Placebo difference scores (∆) for the indices of R–R: mean of R–R intervals; SDNN: standard deviation of R–R intervals; LF: low frequency; HF: high frequency; SampEn: sample entropy; ln (EEG Band Pow): natural logarithm transform of each EEG band power of interest (ϑ, α, β1, β2, and γ) at Fz, Cz, and Pz recording sites.

For the self-pain condition, each of these separated PCAs (varimax rotation) yielded a three orthogonal factors solution (eigenvalues > 1) that were exported as standardized factor scores and used for the correlation analyses. In terms of HRV changes, common to all these analyses was the first factor loading on frequency domain HRV difference scores (∆) that we labeled as "S_∆fHRV" (S stands for self-pain). Additionally, we obtained a combined factor loading on ∆SDNN and sample entropy changes that we labeled as "S_∆SDNN & ∆S-Entr." In terms of EEG band power changes, we obtained two factors, one loading on β1 power and the other on β2 power, obtained at midline sites (Fz, Cz, and Pz) that we labeled as "S_∆Midl-β1Pow" and "S_∆Midl-β2Pow". We also obtained the following composite factors, including HRV measures and ϑ, α, and γ power changes: "S_∆tHRV & ∆Midl-ϑPow," "S_∆SDNN & ∆S-Entr & ∆Cz-αPow," "S_∆tHRV & ∆CzPz-αPow," and "S_∆tHRV & ∆CzPz-γPow" (see loadings in boldface reported in the upper section of Table 1). Descriptive statistics for these factors are reported on the left side of Table 2.

**Table 2:** Descriptive statistics for PCA factor difference scores (Control minus Placebo, ∆) including ∆Frequency HRV (∆fHRV), ∆Standard Deviation of R–R time intervals (∆SDNN), ∆Sampling Entropy (∆S-Entr), frequency bands of ϑ, α, β1, β2, and γ (∆Midl-ϑPow, ∆Cz-αPow, ∆CzPz-αPow, ∆Midl-β1Pow, ∆Midl-β2Pow, and ∆CzPz-γPow), respectively for Self-Pain (S) and Other-Pain (O) conditions.

Self-Pain					Other-Pain				
Variable	Mean	Std dev	Min	Max	Variable	Mean	Std Dev	Min	Max
S_∆fHRV	1.25	7.92	− 15.20	18.35	O_∆fHRV	0.87	12.21	− 24.74	55.70
S_∆SDNN & ∆S-Entr	− 6.74	80.61	− 611	119.4	O_∆tHRV & ∆S-Entr	− 4.31	27.19	− 91.10	46.56
S_∆tHRV & ∆Midl-ϑPow	− 9.42	24.23	− 61.70	57.52	O_∆Midl-ϑPow	− 0.53	3.69	− 17.51	8.93
S_∆SDNN & ∆S-Entr & ∆Cz-αPow	− 7.50	67.06	− 512	96.90	O_∆FzCz-αPow	− 3.54	25.72	− 86.40	47.34
S_∆tHRV & ∆CzPz-αPow	− 15.10	58.35	− 125	226.3	O_∆Midl-β1Pow	− 0.167	2.911	− 9.50	8.80
S_∆Midl-β1Pow	2.12	7.01	− 10.4	46.38	O_∆Midl-β2Pow	− 0.26	8.78	− 38.89	31.40
S_∆Midl-β2Pow	3.04	11.91	− 13.8	84.49	O_∆Midl-γPow	0.10	3.31	− 12.10	11.16
S_∆tHRV & ∆CzPz-γPow	− 17.20	49.58	− 120	117.4	–	–	–	–	–

Similar separate PCAs on physiological difference data performed for the other-pain condition yielded a three orthogonal factors solution. In terms of HRV changes, common to all these analyses was the first factor loading mainly on frequency domain HRV difference scores, and we labeled it as "O_∆fHRV" (O stands for other-pain). We also obtained a combined factor loading on time HRV and sample entropy changes labeled "S_∆tHRV & ∆S-Entr." In terms of EEG band power changes, we obtained four factors loading on ϑ, β1, β2, and γ powers across the three midline sites (Fz, Cz, and Pz) and labeled respectively as "O_∆Midl-ϑPow," "O_∆Midl-β1Pow", "O_∆Midl-β2Pow", and "O_∆Midl- γPow." For the α band, we also obtained a factor including the α power differences at Fz and Cz leads that we labeled as "O_∆FzCz-αPow." All these factors can be derived from loadings in boldface reported in the lower section of Table 1). Descriptive statistics for these factors are reported on the right side of Table 2.

### Statistical analyses

We first calculate partial Pearson correlation coefficients between self-pain and other-pain differences scores (Control minus Placebo), RST-PQ, and ECQ personality traits with change scores on physiological factors. The potential contribution of Gender and State Anxiety difference scores was partially out from these correlations. We also calculated a partial Pearson correlation matrix (gender scores were partially out) among personality traits of interest, including pain and unpleasantness rating difference scores. The probability levels were corrected using the false discovery rate correction (FDR) method^41^ to control false-positive errors. Among the physiological factors significantly correlated with a personality trait, we want to select the best predictors of these traits by avoiding collinearity among them. Thus, we first assess collinearity diagnostics using the Proc Reg procedure available in the SAS-9.4 system. We then solved the collinearity problem by implementing the Elastic Nets method provided by the Proc Glmselect procedure available in the same statistical system. This analysis can overcome the limitations on the variable selection, usually presented in other available similar methods. It can select more than one variable and achieve a better model prediction (see, e.g.^42^). Separately for self-pain and other-pain conditions, we applied the above-described method to select physiological factors as predictors of pain and unpleasantness difference scores (i.e., S_NPDSs and S_NUDSs, O_NPDSs and O_NUDSs). We set a significance level at p = 0.05 after FDR correction. We then tested conditional process models evaluating physiological factors as mediators for the causal influence of the personality traits on S_NPDSs and O_NPDSs. Our choice of personality traits as predictors of pain changes and physiological factors as mediators was due to the conceptual constraint that the mediation models would be reasonable only if the mediator intervened in time between the predictor and outcome^43^, given that personality questionnaires were administered a day before the EEG recordings. We used the PROCESS macro (www.afhayes.com) to perform analyses^44^. We included state anxiety changes (∆STAIY1) and Gender as covariates in these models.

## Results

### Pain and unpleasantness

A repeated measures ANOVA on pain scores of self-pain condition, with Gender as a between and Treatment as a within subjects factor, yielded a main effect for Gender (*F*(1,60) = 4.21, p < 0.05, ɳ^2^_p_ = 0.065) that indicated a higher pain sensation in women compared to men (M = 5.2, SD = 0.95 vs M = 4.7, SD = 1.11). In addition we observed a significant effect for Treatment (*F*(1,60) = 19.92, p < 0.0001, ɳ^2^_p_ = 0.249) indicating Placebo treatment was effective in pain reduction (M = 5.2, SD = 1.18 vs M = 4.7, SD = 1.15). The interaction of Gender by Treatment was not significant (*F*(1,60) = 1.14, p = 0.290, ɳ^2^_p_ = 0.019). The ANOVA on unpleasantness scores provided a significant effect for Treatment (*F*(1,60) = 8.94, p < 0.01, ɳ^2^_p_ = 0.130), showing a lower unpleasantness to Placebo as compared to Control treatment (M = 4.8, SD = 1.30 vs M = 4.4, SD = 1.30).

The ANOVA on pain scores for the other-pain condition did not show any significant effect (all *Fs* < 1). A similar analysis on unpleasantness scores of the other-pain condition disclosed a main effect for Treatment (*F*(1,60) = 5.26, p < 0.05, ɳ^2^_p_ = 0.081) which showed a small but significant unpleasantness reduction during Placebo as compared to Control (M = 4.5, SD = 1.28 vs M = 4.9, SD = 1.32, respectively). Descriptive statistics for numerical pain and unpleasantness scores in women and men participants are reported in Table 3.

**Table 3:** Descriptive statistics in women and men participants (1) for the RST-PQ, ECQ, STAI-Y1; (2) for numerical pain and unpleasantness scores: (i) of the Self in the Control (SC-NPS, and SC-NUS), Placebo (SP-NPS, and SP-NUS) treatments and Control minus Placebo difference scores (S_NPDS and S_NUDS); (j) of the Other in the Control (OC-NPS, and OC-NUS), Placebo (OP-NPS, and OP-NUS) treatments and Control minus Placebo difference scores (O_NPDS and O_NUDS).

Variable	Women (N = 32)				Men (N = 30)				Gender	
	Mean	SD	Min	Max	Mean	SD	Min	Max	t(60)	p(FDR)
BAS-TOT	94.1	12.9	71	125	89.3	11.1	67	113	1.58	0.421
BAS-GDP	21.9	3.9	12	28	21.4	2.9	16	28	0.58	0.736
BAS-RI	20.6	4.6	11	28	20.2	4.1	12	28	0.3	0.831
BAS-RR	31.0	4.9	19	39	29.3	4.5	20	39	1.42	0.421
BAS-I	20.7	5.1	3	32	18.4	4.3	10	25	1.94	0.368
BIS	61.3	12.6	42	85	53.5	11.8	29	80	2.52	0.183
FFFS	28.9	4.2	23	37	22.9	6.4	12	37	4.42	0.003
CE	36.3	3.6	27	42	36.6	4.5	24	44	− 0.34	0.831
AE	49.6	9.1	32	64	47.3	7.7	24	60	1.1	0.471
ED	30.2	3.2	23	36	29.0	3.4	20	36	1.4	0.421
TEA	37.5	5.3	28	56	35.8	3.6	29	44	1.47	0.421
CTE	87.4	14.0	29	106	83.0	12.7	36	103	1.31	0.421
STAI-Y1 (C)	36.8	9.8	22	57	33.9	9.3	20	67	1.21	0.461
STAI-Y1 (P)	36.7	11.3	22	72	33.6	10.0	20	59	1.15	0.471
SC-NPS	5.41	1.11	1.54	6.71	5.00	1.23	1.21	6.75	1.37	0.421
SP-NPS	4.99	1.02	2.63	6.79	4.32	1.20	1.46	6.25	2.37	0.042
S_NPDS	0.42	0.96	− 3.09	2.04	0.68	0.99	− 0.83	3.29	− 1.07	0.471
SC-NUS	5.15	1.25	1.21	6.75	4.65	1.47	1.04	6.67	1.46	0.421
SP-NUS	4.74	1.16	2.25	6.29	4.29	1.47	1.00	6.42	1.36	0.421
S_NUDS	0.41	1.22	− 3.12	2.30	0.36	0.72	− 1.17	1.62	0.19	0.880
OC-NPS	4.97	1.06	2.33	6.50	4.83	1.20	2.08	6.50	0.49	0.736
OP-NPS	4.88	1.21	2.25	6.50	4.88	1.29	1.17	6.54	0.00	0.997
O_NPDS	0.09	1.06	− 2.71	2.46	− 0.05	1.04	− 4.05	1.84	0.54	0.736
OC-NUS	4.97	1.18	2.08	6.50	4.80	1.46	1.00	6.83	0.8	0.657
OP-NUS	4.63	1.25	1.83	6.83	4.46	1.33	2.25	6.50	0.51	0.736
O_NUDS	0.34	0.68	− 0.79	2.09	0.34	0.64	− 1.25	1.46	0.6	0.736

Last two columns: T-test values (Women vs Men) and FDR-corrected p values.

*M* Mean, *SD* Standard Deviation, *Min* Minimum, *Max* Maximum, *RST-PQ* Reinforcement Sensistivity Theory-Personality Questionnaire, *BAS-TOT* Behavioral Approach System, *BAS-GDP* Goal-Drive Persistence, *BAS-RI* Reward Interest, *BAS-RR* Reward Reactivity, *BAS-I* Impulsivity, *BIS* Behavioural Inhibition System, *FFFS* Fight-Flight-Freeze System, *C* State Anxiety in the pain control STAI-Y1, *P* placebo STAI-Y1 treatments, *ECQ* Empathy Components Questionnaire, *CE* Cognitive Empathy, *AE* Affective Empathy, *ED* Empathic Drive, *TEA* Total Empathic Ability, *CTE* Cumulative Total Empathy.

A similar ANOVA on rating scores to nonpainful stimulation, separately performed for self-pain and other-pain conditions, did not yield any significant effect involving Treatment (more details are given in Supplement S5).

### Personality scores

In Table 3 are reported descriptive statistics in women and men participants of personality traits and state measures of interest. We also calculated t-tests (FDR correction) between women and men participants for these measures. The FFFS trait was the sole to be significantly higher in women than men.

### Correlations among personality traits and pain rating measures

The partial correlation matrix (the effects of gender were partial out) among personality and pain ratings is reported in Table 4. It is important to note that, among personality traits of interest, FFFS was significantly and negatively correlated with S_NPDS in the self-pain condition (p < 0.05). A post-hoc within-subject t-test disclosed that there was a significant pain reduction in low FFFS scorers (M = 5.1, SD = 1.2 vs M = 4.3, SD = 1.2; t (30) = 4.39, p < 0.001, respectively for Control vs Placebo), whereas in high FFFS scorers pain reduction did not reach the significance level (M = 5.4, SD = 1.2 vs M = 5.1, SD = 1.0; t (30) = 1.93, p = 0.063, respectively). Additionally, in the other-pain condition, TEA was positively correlated with relative placebo induced pain changes (i.e., O_NPDS, p < 0.01, Table 4). A within-subject t-test on O_NPDSs indicated that during PA treatment there was a significant pain decrease in high TEA scorers (M = 5.2, SD = 1.0 vs M = 4.7, SD = 1.3; t (24) = 2.70, p = 0.013, respectively for Control vs Placebo), whereas in low TEA scorers there was no pain reduction (M = 4.7, SD = 1.1 vs M = 4.9, SD = 1.2; t(36) = − 1.41; p = 0.166, respectively).

**Table 4:** Partial correlations for RST-PQ , ECQ personality traits, and Control minus Placebo for State-Anxiety difference scores (∆STAI-Y1), numerical pain and distress rating difference scores, repectively in the self-pain (S_NPDS, S_NUDS) and other-pain conditions (O_NPDS, O_NUDS). The effect of Gender was partial out.

	1	2	3	4	5	6	7	8	9	10	11	12	13	14	15	16	17
1. BAS-TOT	1	0.75^‡^	0.70^‡^	0.76^‡^	0.61^‡^	0.03	− 0.01	0.03	0.33	0.09	0.23	0.21	− 0.25	− 0.02	− 0.03	− 0.08	0.08
2. BAS-GDP		1	0.55^†^	0.51^†^	0.167	− 0.04	− 0.12	0.07	0.17	− 0.02	0.15	0.07	− 0.06	− 0.04	− 0.05	− 0.09	0.02
3. BAS-RI			1	0.30	0.16	− 0.09	− 0.03	0.27	0.15	0.01	0.19	0.12	− 0.08	− 0.14	− 0.13	− 0.08	− 0.12
4. BAS-RR				1	0.31	0.16	0.08	− 0.04	0.43^·^	0.25	0.32	0.40*	− 0.28	0.11	0.01	− 0.05	0.16
5. BAS-I					1	0.02	− 0.01	− 0.19	0.16	0.03	− 0.01	− 0.01	− 0.26	− 0.01	0.09	− 0.03	0.13
6. BIS						1	0.39*	− 0.03	0.06	0.02	0.07	0.08	− 0.10	− 0.04	− 0.01	− 0.05	0.16
7. FFFS							1	0.06	− 0.08	0.07	0.03	0.03	− 0.06	− 0.34*	− 0.14	0.08	− 0.07
8. CE								1	0.20	0.27	0.49^·^	0.44^·^	0.28	− 0.02	− 0.14	0.18	− 0.32
9. AE									1	0.40*	0.56^†^	0.57^†^	− 0.04	− 0.02	0.06	− 0.02	0.03
10. TEA										1	0.41^·^	0.34*	− 0.01	0.01	− 0.02	0.44^·^	− 0.21
11. ED											1	0.59^†^	0.07	0.13	0.09	0.13	− 0.08
12. CTE												1	− 0.08	− 0.02	− 0.03	− 0.07	− 0.09
13. ∆STAIY1													1.00	0.19	0.25	0.09	− 0.15
14. S_NPDS														1	0.74^‡^	− 0.14	0.31
15. S_NUDS															1	− 0.21	0.28
16. O_NPDS																1	0.28
17. O_NUDS																	1

^‡^p < 0.0001; ^†^p < 0.001; ^·^p < 0.01; *p < 0.05; (False Discovery Rate correction).

*RST-PQ* Reinforcement Sensistivity Theory-Personality Questionnaire, *BAS-TOT* Behavioral Approach System, *BAS-GDP* Goal-Drive Persistence, *BAS-RI *Reward Interest, *BAS-RR* Reward Reactivity, *BAS-I* Impulsivity, *BIS* Behavioural Inhibition System, *FFFS* Fight-Flight-Freeze System, *ECQ* Empathy Components Questionnaire, *CE* Cognitive Empathy, *AE* Affective Empathy, *TEA* Total Empathic Ability, *ED* Empathic Drive, *CTE* Cumulative Total Empathy.

### Correlations of physiological factors with personality and pain rating measures

Partial correlations showed that BIS was the only personality trait significantly and positively associated with physiological difference scores of S_∆tHRV & ∆Midl-ϑPow, S_∆tHRV & ∆CzPz-αPow, and S_∆tHRV & ∆CzPz-γPow factors obtained for the self-pain condition (S). These same physiological factors were significantly and negatively associated with S_NPDSs. In addition, S_NUDSs were significantly and negatively correlated with S_∆tHRV & ∆Midl-ϑPow and S_∆tHRV & ∆CzPz-γPow factors (see the left side of Table 5).

**Table 5:** Left-half table: Partial Pearson correlation coefficients of the Behavioral Inhibition System (BIS) personality trait and numerical pain and distress difference scores (Control minus Placebo) in the self-pain (S_NPDS, S_NUDS) condition with HRV and EEG band power difference (∆, Control minus Placebo) factors of interest.

Self-Pain				Other-Pain			
	BIS	S_NPDS	S_NUDS		AE	TEA	O_NPDS
S_∆fHRV	− 0.285	− 0.028	− 0.142	O_∆f HRV	− 0.055	0.201	0.385*
S_∆SDNN & ∆S-Entr	− 0.155	− 0.005	0.004	O_∆tHRV & ∆S-Entr	− 0.374*	− 0.274	0.034
S_∆tHRV & ∆Midl-ϑPow	0.380*	− 0.502^·^	− 0.355*	O_∆Midl-ϑPow	0.001	− 0.287	− 0.422*
S_∆SDNN & ∆S-Entr & ∆Cz-αPow	− 0.128	− 0.038	− 0.017	O_∆FzCz-αPow	0.261	− 0.030	− 0.308
S_∆tHRV & ∆CzPz-αPow	0.397*	− 0.389*	− 0.274	O_∆Midl-β1Pow	− 0.065	0.274	0.323
S_∆Midl-β1Pow	− 0.098	0.301	0.203	O_∆Midl-β2Pow	0.148	0.405*	0.418*
S_∆Midl-β2Pow	− 0.027	0.240	0.169	O_∆Midl- γPow	0.167	0.379*	0.328
S_∆tHRV & ∆CzPz- γPow	0.415*	− 0.472^·^	− 0.329*	–	–	–	–

Right-half table: Partial Pearson correlation coefficients of Affective Empathy (AE), Total Empathy Ability (TEA), and numerical pain difference scores in the other-pain condition (O_NPDS) with physiological difference factor scores showing significant associations. The effect of Gender and State Anxiety changes (Control minus Placebo) was partial out.

^·^p < 0.01; *p < 0.05; (False Discovery Rate correction).

S_: self-pain; S_∆fHRV: frequency HRV difference score; S_∆SDNN & ∆S-Entr: Standard deviation of R–R intervals & Sampling Entropy differences; S_∆tHRV & ∆Midl-ϑPow: R–R time interval & Midline ϑ band power differences; S_∆SDNN & ∆S-Entr & ∆Cz-αPow: Standard deviation of R–R intervals & Sampling Entropy & Central (Cz) α band power differences; S_∆tHRV & ∆CzPz-αPow: R–R time interval & Centro-Parietal (CzPz) α band power differences; S_∆Midl-β1Pow: Midline β1 band power differences; S_∆Midl-β2Pow: Midline β2 band power differences; S_∆tHRV & ∆CzPz- γPow: R–R time interval & Centro-Parietal (Cz and Pz) γ band power differences. O_: other-pain; Labels used for the other-pain conditions follows the same labeling rules used for the self pain.

We found the AE trait significantly and negatively correlated with the O_∆tHRV & ∆S-Entr factor obtained for the other-pain condition. Additionally, TEA trait was positively correlated with O_∆Midl-β2Pow and O_∆Midl-γPow factors, indicating that higher TEA scores were associated with reduced β2 and γ power scores during Placebo relative to Control treatment. Further, O_NPDSs were negatively associated with O_∆fHRV, O_∆Midl-ϑPow, and O_∆Midl-β2Pow (right side of Table 5).

### Self-Pain: physiological factors predictors of BIS, pain, and unpleasantness changes

We want to select physiological factors that better predict BIS scores among the three ones we found significantly correlated with BIS (see the left side of Table 5). We first assess collinearity diagnostics using variance inflation factors *vif*, tolerance *tol*, and collinearity *collin* options of SAS-9.4 regression procedure with the three physiological factors differences as predictors of BIS scores. This analysis suggests excluding the factor S_∆tHRV & ∆CzPz-γPow from further analyses since we found high levels of collinearity of this factor with the other two.

To further combat the multicollinearity, we then tested the multiple regression model using the elastic nets method with Akaike's information selection criterion^45,46^, using as predictors of BIS the two remaining factors. This analysis yielded both factors of S_∆tHRV & ∆Midl-ϑPow and S_∆tHRV & ∆CzPz-αPow as potential predictors of BIS (F(2,59) = 9.01, p < 0.01, ɳ^2^_p_ = 0.190; R-Square = 0.234; Glmselect procedure, SAS-9.4^47^).

Further, to select and avoid multicollinearity among the three physiological measures, we found significantly correlated with self-pain change scores (S_NPDS, see the left side of Table 5), those that best predict S_NPDSs, we used the same multiple regression procedure reported above, including multiple regression and the Elastic Nets method with Akaike's information selection criterion^45,46^. This method yielded the S_∆tHRV & ∆Midl-ϑPow factor as the most reliable predictor of S_NPDS (*F*(1,60) = 23.16, p < 0.001, ɳ^2^_p_ = 0.278; R-Square = 0.279). More details are reported in Supplements S6-1, S6-2, and S6-3.

A similar method used for the selection, among two potential physiological predictors of unpleasantness reduction scores (S_NUDSs; see left side of Table 5) yielded again the S_∆tHRV & ∆Midl-ϑPow as the sole reliable predictor of S_NUDS (*F*(1,60) = 8.28, p < 0.01, ɳ^2^_p_ = 0.120; R-Square = 0.121).

### Personality and Physiological influence on Placebo induced self-pain changes

**Figure 4:**
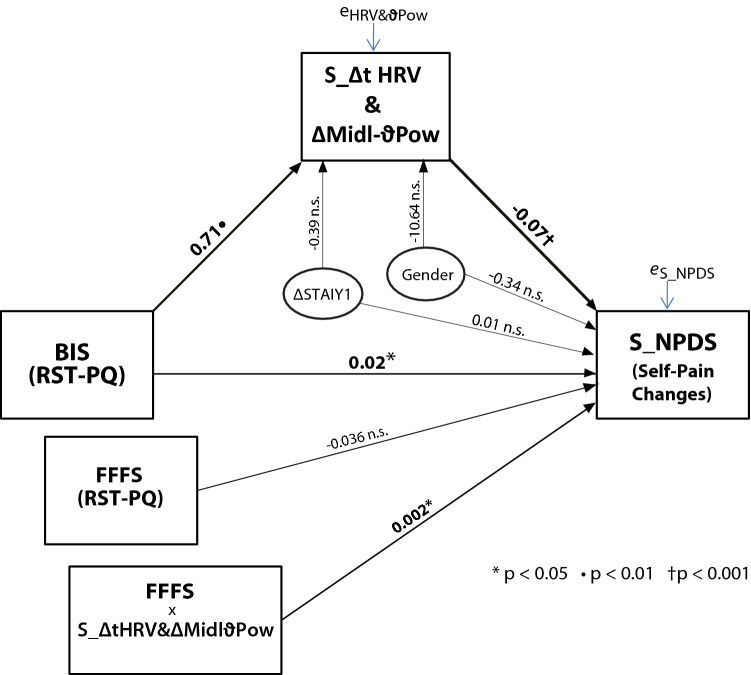
Conditional process model depicting the causal influence of BIS on the outcome S_NPDS with the "S_∆tHRV & ∆Midl-ϑPow" factor as a potential mediator and with FFFS as a moderator of the S_∆tHRV & ∆Midl-ϑPow influence on S_NPDS. The thickness of the arrows indicates the strength of the link between variables. Gender and State-Anxiety changes (∆STAIY1) are included as covariates.

The S_∆tHRV & ∆Midl-ϑPow factor was significantly correlated with both BIS and S_NPDS (left side of Table 5), BIS was significantly correlated with FFFS and the latter with S_NPDSs (Table 4). Starting from these observations, we tested a conditional process model by entering S_∆tHRV & ∆Midl-ϑPow as a potential mediator of the causal influence of BIS on the outcome S_NPDS combined with FFFS as a moderator of the S_∆tHRV & ∆Midl-ϑPow influence on S_NPDS (pp. 338–354; model 14)^44^. The model included ∆STAIY1 and Gender as covariates (see the upper-half section in Table 6 and Fig. 4). The total effect of BIS on S_NPDSs (including the mediating role of S_∆tHRV & ∆Midl-ϑPow and the moderating effect of FFFS) was significant (p < 0.0001), as well as the direct effect of BIS on S_NPDSs (effect = 0.021, t = 2.344, p = 0.0227, LLCI = 0.003, ULCI = 0.039). In addition, we did not detect any significant effect of Gender and ∆STAIY1 on the S_∆tHRV & ∆Midl-ϑPow and S_NPDS outcomes (Table 6 and Fig. 4). We obtained bootstrap confidence intervals of the conditional indirect effects of BIS on S_NPDS through the S_∆tHRV & ∆Midl-ϑPow factor at values of the moderator FFFS corresponding to the 10th, 25th, 50th, 75th, and 90th percentile of the distribution of FFFS. Bias-corrected bootstrap confidence intervals are based on 10,000 bootstrap samples. This effect was significant and negative for FFFS scores of 17, 21, 25, and 29, corresponding to the 10th, 25th, 50th, 75th percentile. For each of these FFFS scores, the bootstrap confidence interval [BootLLCI, BootULCI] was entirely below zero ([− 0.061, − 0.010], [− 0.050, − 0.009], [− 0.041, − 0.007], [− 0.033, − 0.004], respectively). The conditional process model is depicted in Fig. 4.

**Table 6:** Upper quadrant—Model-1: conditional process analysis using "S_∆tHRV & ∆Midl-ϑPow" as a potential mediator of the causal influence of BIS on the outcome of self-pain changes (S_NPDS) combined with FFFS as a moderator of the S_∆tHRV & ∆Midl-ϑPow influence on S_NPDS.

Model-1	Self-Pain: Control minus Placebo difference scores (S_NPDS)											
	Outcome: S_∆t HRV & ∆Midl-ϑPow						Outcome: S_NPDS					
	coeff	se	t	p	LLCI	ULCI	coeff	se	t	p	LLCI	ULCI
**Predictors**												
Constant	− 45.102	14.428	− 3.126	0.003	− 73.983	− 16.221	0.107	0.722	0.147	0.883	− 1.341	1.554
S_∆t HRV & ∆Midl-ϑPow	–	–	–	–	–	–	− 0.072	0.020	− 3.531	0.001	− 0.113	− 0.031
BIS	0.711	0.227	3.133	0.003	0.257	1.166	0.021	0.009	2.344	0.023	0.003	0.039
FFFS	–	–	–	–	–	–	− 0.036	0.021	− 1.677	0.099	− 0.079	0.007
S_∆t HRV & ∆Midl-ϑPow x FFFS	–	–	–	–	–	–	0.002	0.001	2.456	0.017	0.000	0.003
**Covariates**												
Gender	− 10.647	5.707	− 1.866	0.067	− 22.070	0.776	− 0.340	0.226	− 1.503	0.139	− 0.793	0.113
∆STAIY1	− 0.391	0.392	− 0.999	0.322	− 1.175	0.393	0.012	0.014	0.844	0.402	− 0.016	0.040
	**R-sq = **0.262, **F**(3, 58) = 6.861, p = 0.0005						**R-sq = **0.465, **F**(6, 55) = 7.950, p < 0.0001					

Model-2	Other-Pain: Control minus Placebo difference scores (O_NPDSs)											
	Outcome: O_∆Midl-β2Pow						Outcome: Other-Pain Reduction (O_NPDS)					
	coeff	se	t	p	LLCI	ULCI	coeff	se	t	p	LLCI	ULCI
**Predictors**												
Constant	− 30.726	8.754	− 3.510	0.001	− 48.250	− 13.202	− 2.653	1.088	− 2.439	0.018	− 4.832	− 0.475
Total Empathic Ability (TEA)	0.778	0.230	3.377	0.001	0.317	1.239	0.075	0.028	2.625	0.011	0.018	0.132
O_∆Midl-β2Pow	–	–	–	–	–		0.034	0.015	2.297	0.025	0.004	0.064
**Covariates**												
Gender	4.009	2.105	1.905	0.062	− 0.205	8.222	− 0.11	0.245	− 0.451	0.654	− 0.601	0.38
∆STAIY1	0.000	0.148	− 0.002	0.998	− 0.297	0.297	0.014	0.017	0.834	0.408	− 0.02	0.048
	**R-sq = **0.184, **F**(3, 58) = 4.364, p = 0.008						**R-sq = **0.273, **F**(4, 57) = 5.358, p = 0.001					

Bottom quadrant: Model-2: simple mediation model testing the contribution of Total Empathic Ability (TEA) as a potential causal factor influencing changes in other-pain rating scores (O_NPDS) through its indirect influence of O_ΔMidl-β2Pow factor as a mediator causing other-pain changes as the final consequent. Gender and State-anxiety differences (ΔSTAIY1) are entered as covariates in both models.

### Other-Pain: physiological factors predictors of TEA and placebo pain changes

We found two physiological factors that were significantly correlated with TEA scores (i.e., O_∆Midl-β2Pow and O_∆Midl-γPow; see the right half side of Table 5). We used the same multiple regression procedure reported above to select the best TEA predictors and avoid multicollinearity. We also used the Elastic Nets method with Akaike's information selection criterion^45,46^ to further control multicollinearity. Both analyses selected the O_∆Midl-β2Pow variable as the most reliable predictor of TEA (F(1,60) = 7.46, p < 0.01, ɳ^2^_p_ = 0.120; R-Square = 0.111; for more details see Tables S6-4 in Supplement).

We found three physiological factors, namely O_∆f HRV, O_∆Midl-ϑPow, and O_∆Midl-β2Pow, that were significantly correlated with O_NPDSs (right half of Table 5). To select among these three factors those that better predict O_NPDS scores and to detect collinearity, we used the same collinearity diagnostic method described above. We found that collinearity among these variables was not significant. Additionally, a multiple regression using the Elastic Nets method with Akaike’s information selection criterion retained all the three physiological factors as reliable predictors of O_NPDSs (F(3,58) = 4.80, p < 0.01, ɳ^2^_p_ = 0.199; R-Square = 0.198; see Tables S6-5 in Supplement).

### Personality and Physiological influences on Placebo induced other-pain changes

**Figure 5:**
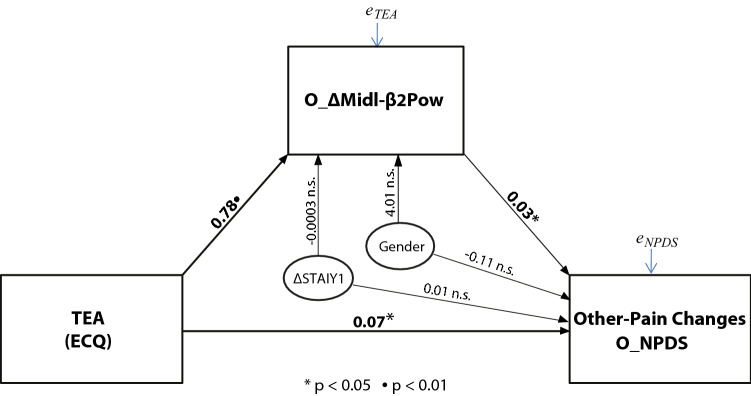
Simple mediator model testing the Total Empathy Ability trait (TEA) as the independent variable influencing other-pain changes (O_NPDS) through the mediation of midline β2 (22–32 Hz, 100–180 ms) power factor changes "O_∆Midl-β2Pow" induced by PA treatment in the other-pain condition. Direct and indirect effects (Ind) are reported. The thickness of the arrows indicates the strength of the link between variables. Gender and State-Anxiety changes (∆STAIY1) are entered as covariates.

Considering that the three variables of O_∆Midl-β2Pow, O_NPDS, and TEA were all significantly correlated between them (see Table 4 and right half of Table 5), we used a simple mediation model to test the causal effect of the TEA trait on O_NPDS through the O_∆Midl-β2Pow factor as a potential mediator causing O_NPDS as the final consequent^44^ (pp. 85–122; model 4). We included state anxiety changes (∆STAIY1) in the model and Gender as covariates. This mediation analysis disclosed that direct and indirect effects were significant (see the lower-half section of Table 6 and Fig. 5). The total effect of TEA on O_NPDSs was significant (effect = 0.101, t = 3.75, p = 0.0004, LLCI = 0.047, UCLI = 0.155), as well as the direct effect of TEA on O_NPDSs (effect = 0.075, t = 2.62, p = 0.011, LLCI = 0.018, UCLI = 0.132) and its indirect effect through the O_∆Midl-β2Pow factor (effect = 0.027, Boot SE = 0.018, BootLLCI = 0.000, BootUCLI = 0.072). This model indicated that higher TEA scores directly produced relatively higher positive O_NPDSs, i.e., relatively smaller pain sensations during Placebo treatment. The indirect positive effect of the O_∆Midl-β2Pow factor mediates this influence (Fig. 5). In addition, we did not detect significant effects for the covariates Gender and ∆STAIY1 on the O_NPDS outcome.

## Discussion

One of the main aims of the present study was to manipulate the first-hand experience of pain, devoted to enhancing expectation for pain reduction, and test whether this experience also affects empathy for pain. Current results disclosed that PA treatment (i.e., a compound of manipulation of pain sensation and verbal suggestion inducing PA) effectively reduced both first-hand pain and unpleasantness sensations, with women experiencing higher sensitivity to nonpainful and painful stimulation levels than men. However, the phenomenon of empathic analgesia was not found significant for the other-pain scores, although we saw it as effective, albeit weakly, in reducing other unpleasantness. Additionally, after controlling for Gender, we did find a significant negative association between FFFS trait (but not BIS) and self-pain reduction during PA. This effect indicated that Placebo treatment in low FFFS participants effectively reduced pain sensation, whereas pain reduction did not reach the significance level in high FFFS participants. The fact that we failed to find a significant correlation between self-pain reduction and BIS, while, on the other hand, this relation was substantial for FFFS, is not surprising. Indeed, observations in earlier studies showed that subjects higher in dispositional pain-related fear had reduced PA reponding^23,48–51^. Mostly, there is experimental evidence in these participants that the anticipation of a painful shock increases subjective fear^52^. This finding demonstrates a substantial impairment of mechanisms underlying PA in high FFFS individuals who are highly disposed to fear, mainly when an anticipatory cue, indicating that they would receive a painful electric shock, induces a potential fear.

Further, we found a significant positive link between TEA trait and other-pain rating changes (Table 4), demonstrating a considerable pain decrease after PA treatment in high TEA but not in low TEA scorers. Unpleasantness did not show substantial differences in high TEA scorers. Interestingly, these findings align with Singer and Lamm's suggestions^53^ that empathy is a highly flexible phenomenon influenced by several factors as stable empathy traits of the empathizer and its interpersonal relationship with the other. Our observations indicate that individual differences in empathic ability traits can account for empathy for pain.

In sum, the present self-pain findings parallel those previously observed by Rutgen et al.^27^, whereas our other-pain rating finding aligns with Rutgen et al.'s one only in higher TEA trait individuals. We did not detect any significant effect of situational empathy for the emotional component of other pain, i.e., when participants evaluate the unpleasantness of the other. Research using ERP and startle data has shown that the induction of fear completely abolished the effect of the placebo intervention^23,51,54^. Thus, we think that fear of pain may have reduced the placebo effect, especially for empathic pain and unpleasantness.

Self-pain reduction correlated negatively with changes in three physiological factors. Each of these associations indicated that higher self-pain reduction was associated with (1) longer R–R time intervals (time-HRV) and (2) reduced midline ϑ-band activity, (3) enhanced centroparietal α activity, (4) reduced centroparietal γ activity (left side of Table 5). These observations corroborate and extend previous EEG research findings that phasic pain stimuli suppress α oscillations^55,56^, enhancing ϑ activity^57^ and γ activity^58^. However, multiple regression analyses using the elastic nets method yielded a factor encompassing higher R–R time intervals and reduced midline EEG ϑ activity as the only effective capture of meaningful information in predicting placebo pain reduction. This finding extends original findings linking HR dynamics with attention and mood during stress^59^ and our PA findings obtained for tonic pain^28^. Our observation of a joint covariation of enhanced R–R time interval and reduced ϑ activity aligns with growing research suggesting that HRV reflects the brain–heart interaction^60^. Notably, Thayer and colleagues' findings conceptualized a neurovisceral model of emotion regulation^61^, suggesting an essential link between cognitive performance, HRV, and prefrontal neural function that is important for physical health and mental stability. In line with this model, our present findings suggest that pain relief is consequent to the activation of the parasympathetic system rather than reduced activation of the sympathetic system, given that vagal influences on cardiac control are much faster than sympathetic ones. The organism facilitates higher self-regulation of pain/distress control when the immediate vagal effect is enhanced. Additionally, we obtained significant associations of self-pain relief with relatively higher EEG α and smaller γ power (see the left half of Table 5). We think that these current findings complement previous reports showing that brief noxious stimuli induce a complex spectral spatial–temporal response pattern characterized by three primary frequency responses: ϑ^62^, α^63^, fast-β, and γ^64^ in the suprasylvian region and ACC along with the frontoparietal operculum and insula. However, the present study indicated that the factor including the covariation of ϑ and time-HRV changes was the most reliable predictor of Placebo pain relief. This finding adds new information and parallels previous observations suggesting that enhanced ϑ-band (4–7 Hz) and γ-band (> 28 Hz) activities are likely expressions of prestimulus^65^ and consequent stimulus pain processing^66^.

Finally, conditional process analysis disclosed that the BIS trait was significantly linked to self-pain reduction through the mediating effect of ∆tHRV & ∆Midl-ϑPow that was moderated by FFFS. These findings indicate that lower BIS scorers would show higher PA pain reduction partly because of the boost of HR slowing and reduced midline ϑ activity, which enhanced the placebo analgesic effect. However, the link between BIS through the ∆tHRV & ∆Midl-ϑPow on self-pain reduction would be more accentuated among lower FFFS participants (Tables 1, 6, and Fig. 4). As far as we know, this is the first study disclosing the conditional BIS's influence on PA responding through the indirect mediating effect of HR slowing moderated by the FFFS trait. These findings linking both BIS and FFFS traits with HRV and EEG ϑ activity on PA responding are new and merit to be discussed. We think at least two reasons may account for the lack of research on FFFS as a modulator of PA responding. The first reason is that BIS has usually been measured using the BIS/BAS questionnaire^67^, which includes items of both the FFFS and BIS but does not directly measure the FFFS^68^.

The inclusion of active and passive avoidance items into the BIS may account for inconsistent findings in studies relating the BIS scale to placebo and nocebo effects^69^. The second reason is that most previous studies reporting a relationship between BIS and PA use tonic pain stimulation rather than phasic stimulation (for review, see^70^), and research using a phasic stimulus did not provide trait measures related to fear. In the present study, a visual cue anticipated each painful electric stimulus (i.e., an orange spark delivered on 5 s before delivering a pain stimulus, see Fig. 1) that may have induced participants to activate both fear of pain and pain to the painful stimulus onset. Since we found an inverse relation between FFFS scores and PA responses, we think that this finding complement Peter Lyby and colleagues' findings that higher fear of pain trait reduces placebo analgesic responding^51^ and later findings that induced fear abolished a weak PA and most pronounced in subjects who were highest in measures of fear of pain. Mainly, our current new finding aligns with the rRST conceptualization that BIS (anxiety) and FFFS (fear) are separated systems governing defensive behavior^71^. Some research has suggested that the 'direction' of defensive behavior can distinguish FFFS from BIS^72^. The FFFS is active with avoidance of the threatening stimulus (defensive avoidance), while the BIS is active when the threatening stimulus is met (defensive approach). If the situation requires an attack on the threat (fight), both the BIS and the FFFS are activated. Our current finding linking a reduction of EEG ϑ to PA with lower BIS is in line with the original and more recent J. A. Gray conceptualization^16^, suggesting that activation of the BIS generates a particular EEG rhythm in the septohippocampal system (SHS), namely the ϑ rhythm. More recent experimental work supports the association of higher ϑ power reactivity with response execution during goal conflict in higher BIS participants^21^.

In terms of ECQ personality traits, simple mediation analysis highlighted that higher empathic ability (TEA) scores directly influenced more pronounced other-pain reduction and indirectly through the positive mediation of midline β2 power changes (Table 6 and Fig. 5). Since we found that different physiological factors mediated the influence of personality traits on self-pain and other-pain reductions (see Figs. 4 and 5), we believe that the placebo effect on empathic analgesia engages top-down modulated neural processes functionally different from those committed by the first-hand PA treatment. These are new findings, considering that the available research evidence does not yet allow a more precise assignment of the different components to the various modulations of pain (see Ploner et al.’s review^73^). Additionally, the present PA empathy findings seem to be compatible with previous findings showing that the observation of other's pain increased activation in the sensorimotor cortex, as expressed by increased central β (13–30 Hz)^10^ activity, reflecting an increased readiness for a defensive motor reaction of active avoidance (fear) or escape behavior^12^.

### Limitations

The present study has some limitations that deserve consideration. First, the current findings cannot generalize to the clinical population since we obtained them from healthy and young participants. Mainly, our electrophysiological correlations may not be paralleled by placebo analgesia findings derived from pain patients or participants who have suffered severe or chronic pain^74^. Second, we administered the ECQ alone and missed using an available scale specifically developed to measure empathy for others' pa^75^. Third, in this study, we have provided measures of empathic ability traits derived from previous psychological and neuroscience research, ignoring that empathy results from a complex process requiring several intermediate processing stages. This limitation makes it difficult to determine the locus of any effect that influences the empathic response. We agree with the alternative proposal by Coll and collaborators^76^ explaining empathic response as individual differences in 'emotion identification' (i.e., the ability to identify another's emotional state) and the degree to which the identification of another's emotional state causes' affect sharing' in the self. This approach may account for mixed results from previous research concerning the effects of empathy on information processing^77^. In sum, future research should point to (1) generalizing experimental findings into clinical application, (2) understanding how event-related brain- oscillations changes relate to higher-order empathic responses (i.e., emotion identification, affective sharing, and emotion regulation), and how empathic responses promoted approach-related prosocial behaviors.

## Supplementary Information

Supplementary Information.

## References

[1] Betti V, Aglioti SM (2016). Dynamic construction of the neural networks underpinning empathy for pain. Neurosci. Biobehav. Rev..

[2] Rizzolatti G, Sinigaglia C (2010). The functional role of the parieto-frontal mirror circuit: Interpretations and misinterpretations. Nat. Rev. Neurosci..

[3] Cheng Y, Yang C-Y, Lin C-P, Lee P-L, Decety J (2008). The perception of pain in others suppresses somatosensory oscillations: A magnetoencephalography study. Neuroimage.

[4] Perry A, Bentin S, Bartal IB-A, Lamm C, Decety J (2010). "Feeling" the pain of those who are different from us: Modulation of EEG in the mu/alpha range. Cogn. Affect. Behav. Neurosci..

[5] Hoenen M, Lübke KT, Pause BM (2015). Somatosensory mu activity reflects imagined pain intensity of others. Psychophysiology.

[6] Motoyama Y, Ogata K, Hoka S, Tobimatsu S (2017). Frequency-dependent changes in sensorimotor and pain affective systems induced by empathy for pain. J. Pain Res..

[7] Avenanti A, Bueti D, Galati G, Aglioti SM (2005). Transcranial magnetic stimulation highlights the sensorimotor side of empathy for pain. Nat. Neurosci..

[8] Avenanti A, Minio-Paluello I, Sforza A, Aglioti SM (2009). Freezing or escaping? Opposite modulations of empathic reactivity to the pain of others. Cortex.

[9] Avenanti A, Sirigu A, Aglioti SM (2010). Racial bias reduces empathic sensorimotor resonance with otheR–Race pain. Curr. Biol..

[10] Riečanský I, Paul N, Kölble S, Stieger S, Lamm C (2015). Beta oscillations reveal ethnicity ingroup bias in sensorimotor resonance to pain of others. Soc. Cogn. Affect. Neurosci..

[11] Riečanský I, Lamm C (2019). The role of sensorimotor processes in pain empathy. Brain Topogr..

[12] Riečanský I, Lengersdorff LL, Pfabigan DM, Lamm C (2020). Increasing self-other bodily overlap increases sensorimotor resonance to others' pain. Cogn. Affect. Behav. Neurosci..

[13] Benedetti F, Mayberg HS, Wager TD, Stohler CS, Zubieta JK (2005). Neurobiological mechanisms of the placebo effect. J. Neurosci..

[14] Hoffman GA, Harrington A, Fields HL (2005). Pain and the Placebo: What we have learned. Perspect. Biol. Med..

[15] Eisenberger NI (2015). Social pain and the brain: Controversies, questions, and where to go from here. Annu. Rev. Psychol..

[16] Gray JA, McNaughton N (2000). The Neuropsychology of Anxiety: An Enquiry into the Functions of the Septo-hippocampal System.

[17] Corr PJ, McNaughton N (2012). Neuroscience and approach/avoidance personality traits: A two stage (valuation–motivation) approach. Neurosci. Biobehav. Rev..

[18] McNaughton N, Corr PJ (2008). The Reinforcement Sensitivity Theory of Personality.

[19] Corr PJ, Cooper AJ (2016). The reinforcement sensitivity theory of personality questionnaire (RST-PQ): Development and validation. Psychol. Assess..

[20] Moore RA, Gale A, Morris PH, Forrester D (2006). Theta phase locking across the neocortex reflects cortico-hippocampal recursive communication during goal conflict resolution. Int. J. Psychophysiol..

[21] Moore RA, Mills M, Marshman P, Corr PJ (2012). Behavioural inhibition system (BIS) sensitivity differentiates EEG theta responses during goal conflict in a continuous monitoring task. Int. J. Psychophysiol..

[22] Trenado C, Pedroarena-Leal N, Cif L, Nitsche M, Ruge D (2018). Neural oscillatory correlates for conditioning and extinction of fear. Biomedicines.

[23] Lyby PS, Aslaksen PM, Flaten MA (2011). Variability in placebo analgesia and the role of fear of pain: An ERP study. Pain.

[24] Vecchio A, De Pascalis V (2021). ERP indicators of self-pain and other pain reductions due to placebo analgesia responding: The Moderating role of the fight-flight-freeze system. Brain Sci..

[25] Coen SJ (2011). Neuroticism influences brain activity during the experience of visceral pain. Gastroenterology.

[26] Kumari V, Das M, Wilson GD, Goswami S, Sharma T (2007). Neuroticism and brain responses to anticipatory fear. Behav. Neurosci..

[27] Rütgen M, Seidel EM, Riečanský I, Lamm C (2015). Reduction of empathy for pain by placebo analgesia suggests functional equivalence of empathy and first-hand emotion experience. J. Neurosci..

[28] De Pascalis V, Scacchia P (2019). The influence of reward sensitivity, heart rate dynamics and EEG-delta activity on placebo analgesia. Behav. Brain Res..

[29] Batchelder L, Brosnan M, Ashwin C (2017). The development and validation of the empathy components questionnaire (ECQ). PLoS ONE.

[30] Spielberger CD, Gorsuch R, Lushene R, Vagg PR, Jacobs GA (1988). Manual for the statetrait anxiety inventory (form Y).

[31] Singer T (2004). Empathy for pain involves the affective but not sensory components of pain. Science.

[32] Katsarava ZA (2006). A novel method of eliciting pain-related potentials by transcutaneous electrical stimulation. Headache.

[33] Cruccu G (2000). Conduction velocity of the human spinothalamic tract as assessed by laser evoked potentials. NeuroReport.

[34] Perchet C (2012). Do we activate specifically somatosensory thin fibres with the concentric planar electrode? A scalp and intracranial EEG study. Pain.

[35] Donchin E (1981). Surprise!… Surprise?. Psychophysiology.

[36] Kirsch I, Weixel LJ (1988). Double-blind versus deceptive administration of a placebo. Behav. Neurosci..

[37] Price DD (1999). An analysis of factors that contribute to the magnitude of placebo analgesia in an experimental paradigm. Pain.

[38] Delorme A, Sejnowski T, Makeig S (2007). Enhanced detection of artifacts in EEG data using higher-order statistics and independent component analysis. Neuroimage.

[39] McFarland DJ, McCane LM, David SV, Wolpaw JR (1997). Spatial filter selection for EEG-based communication. Electroencephalogr. Clin. Neurophysiol..

[40] Tarvainen MP, Niskanen J-P, Lipponen JA, Ranta-Aho PO, Karjalainen PA (2014). Kubios HRV–heart rate variability analysis software. Comput. Methods Programs Biomed..

[41] Benjamini Y, Hochberg Y (1995). Controlling the false discovery rate: A practical and powerful approach to multiple testing. J. R. Stat. Soc. B.

[42] Zou H, Hastie T (2005). Regularization and variable selection via the elastic net. J. R. Stat. Soc. B.

[43] Tate CU (2015). On the overuse and misuse of mediation analysis: It may be a matter of timing. Basic Appl. Soc. Psychol..

[44] Hayes AF (2013). Introduction to Mediation, Moderation, and Conditional Process Analysis: A Regression-Based Approach.

[45] Mallows CL (1973). Some comments on Cp. Technometrics.

[46] Hocking RR (1976). A Biometrics invited paper: The analysis and selection of variables in linear regression. Biometrics.

[47] Schreiber-Gregory DN (2018). Ridge Regression and multicollinearity: An in-depth review. Model. Assist. Stat. Appl..

[48] Aslaksen PM, Bystad M, Vambheim SM, Flaten MA (2011). Gender differences in placebo analgesia: Event-related potentials and emotional modulation. Psychosom. Med..

[49] Flaten MA, Aslaksen PM, Lyby PS, Bjørkedal E (2011). The relation of emotions to placebo responses. Philos. Trans. R. Soc. B.

[50] Johansen O, Brox J, Flaten MA (2003). Placebo and nocebo responses, cortisol, and circulating beta-endorphin. Psychosom. Med..

[51] Lyby PS, Aslaksen PM, Flaten MA (2010). Is fear of pain related to placebo analgesia?. J. Psychosom. Res..

[52] Phelps EA (2001). Activation of the left amygdala to a cognitive representation of fear. Nat. Neurosci..

[53] Singer T, Lamm C (2009). The social neuroscience of empathy. Ann. N. Y. Acad. Sci..

[54] Lyby PS, Forsberg JT, Åsli O, Flaten MA (2012). Induced fear reduces the effectiveness of a placebo intervention on pain. Pain.

[55] Mouraux A, Guerit J, Plaghki L (2003). Non-phase locked electroencephalogram (EEG) responses to CO2 laser skin stimulations may reflect central interactions between A∂-and C-fibre afferent volleys. Clin. Neurophysiol..

[56] Hu L, Peng W, Valentini E, Zhang Z, Hu Y (2013). Functional features of nociceptive-induced suppression of alpha band electroencephalographic oscillations. J. Pain.

[57] Russ MJ, Campbell SS, Kakuma T, Harrison K, Zanine E (1999). EEG theta activity and pain insensitivity in self-injurious borderline patients. Psychiatry Res..

[58] Schulz E, Zherdin A, Tiemann L, Plant C, Ploner M (2011). Decoding an individual's sensitivity to pain from the multivariate analysis of EEG data. Cereb. Cortex.

[59] Young H, Benton D (2015). We should be using nonlinear indices when relating heart-rate dynamics to cognition and mood. Sci. Rep..

[60] Porges SW (1995). Cardiac vagal tone: a physiological index of stress. Neurosci. Biobehav. Rev..

[61] Thayer JF, Lane RD (2000). A model of neurovisceral integration in emotion regulation and dysregulation. J. Affect. Disord..

[62] Garcia-Larrea L, Frot M, Valeriani M (2003). Brain generators of laser-evoked potentials: From dipoles to functional significance. Clin. Neurophysiol..

[63] Ploner M, Gross J, Timmermann L, Pollok B, Schnitzler A (2006). Pain suppresses spontaneous brain rhythms. Cereb. Cortex.

[64] Gross J, Schnitzler A, Timmermann L, Ploner M (2007). Gamma oscillations in human primary somatosensory cortex reflect pain perception. PLoS Biol..

[65] Taesler P, Rose M (2016). Prestimulus theta oscillations and connectivity modulate pain perception. J. Neurosci..

[66] Schulz E (2015). Prefrontal gamma oscillations encode tonic pain in humans. Cereb. Cortex.

[67] Carver CS, White TL (1994). Behavioral inhibition, behavioral activation, and affective responses to impending reward and punishment: The BIS/BAS scales. J. Pers. Soc. Psychol..

[68] Heym N, Ferguson E, Lawrence C (2008). An evaluation of the relationship between Gray's revised RST and Eysenck's PEN: Distinguishing BIS and FFFS in Carver and White's BIS/BAS scales. Pers. Individ. Differ..

[69] Corsi N, Colloca L (2017). Placebo and nocebo effects: The advantage of measuring expectations and psychological factors. Front. Psychol..

[70] Kennis M, Rademaker AR, Geuze E (2013). Neural correlates of personality: An integrative review. Neurosci. Biobehav. Rev..

[71] Blanchard DC, Hynd AL, Minke KA, Minemoto T, Blanchard RJ (2001). Human defensive behaviors to threat scenarios show parallels to fear- and anxiety-related defense patterns of non-human mammals. Neurosci. Biobehav. Rev..

[72] McNaughton N, Corr PJ (2004). A two-dimensional neuropsychology of defense: Fear/anxiety and defensive distance. Neurosci. Biobehav. Rev..

[73] Ploner M, Sorg C, Gross J (2017). Brain rhythms of pain. Trends Cogn. Sci..

[74] Petrini L, Arendt-Nielsen L (2020). Understanding pain catastrophizing: Putting pieces together. Front. Psychol..

[75] Giummarra MJ (2015). Affective, sensory and empathic sharing of another's pain: The empathy for pain scale. Eur. J. Pain.

[76] Coll MP (2017). Are we really measuring empathy? Proposal for a new measurement framework. Neurosci. Biobehav. Rev..

[77] Fabi S, Leuthold H (2017). Empathy for pain influences perceptual and motor processing: Evidence from response force, ERPs, and EEG oscillations. Soc. Neurosci..

