# Supplementary material for: The influence of EEG oscillations, heart rate variability changes, and personality on self-pain and empathy for pain under placebo analgesia
Source: Sci Rep. 2022 Apr 11;12:6041. doi: 10.1038/s41598-022-10071-9 (PMC9001726; doi:10.1038/s41598-022-10071-9)
Supplement: Supplementary file 1 — Supplementary Information. [file 41598_2022_10071_MOESM1_ESM.docx]

**Supplementary Information**

**The influence of EEG Oscillations, Heart Rate Variability changes, and Personality on Self-Pain and Empathy for Pain under Placebo Analgesia**

Vilfredo De Pascalis, Arianna Vecchio

Sapienza University of Rome, Italy

**Section S1**

**The study and Participants.** This study is a secondary analysis of behavioral and raw EEG data collected to publish the primary findings of our parent study on PA^1^. In the current work, we focused on the relationship between induced oscillatory EEG responses and HRV indexes of self-pain and other pain changes due to PA responding by considering the role of motivational and empathy traits.

Initially, we recruited 83 participants for this study. However, only 63 participants were available for data analyses due to technical problems during physiological recording. Participants were neurotypical right-handed university student volunteers, aged between 18 and 29 years (32 women: M = 21.56, SD = 2.4, men: M = 23.03, SD = 2.63) volunteered to participate. The Italian adaptation of the Edinburgh Handedness Inventory^2^ measured participants’ handedness. All participants were medication-free and had no reported history of using painkillers, corticosteroids, or psychotropic substances. They did not declare color-blindness and psychiatric or neurological illness. For the EEG recording session, we required women participants to be between the 5th and 11th day after the onset of their menses. This restriction avoided possible variations on EEG recordings due to the menstrual period^3^. No ingestion of coffee and smoking for at least two hours before the EEG session was recommended^4^. The experimental protocol was conducted under the Helsinki Declaration (1964) and approved by the Institutional Review Board (IRB) of the Department of Psychology of Sapienza University of Rome (protocol number 0001291 issued on 07/12/2017). Participants gave their written consent to participate in the study and completed a set of trait and state questionnaires. We did not provide participants with any specific hypothesis concerning self-report measures and placebo in the information sheet or verbal communication. We determined sample size through an *a priori* power analysis for *f* = 0.40, two-tailed, α = 0.05, power = 0.80 we obtained a value of N=52.

**Detection of potential outliers.** We calculated Cook’s Distance^5^ (CD; Cook, 1977) to detect potential outliers. In multivariate space, Cook’s distance for the ith observation reflects the differences between the predicted responses from the model constructed from all the data and the predicted responses from the model constructed by excluding the *i*th observation. A larger CD value indicates a more influential subject. The conventional cut-off point for determining an influential observation is 4/*N* (in our case: N= 63, CD = 0.063). When a case had a Cook’s Distance that exceeded this threshold (the highest Cook’s Distance detected on any analysis was 0.52), we excluded this case and re-processed the data. We excluded only one male participant in our results. Thus, we performed data analyses for only 62 participants (32 women).

**Questionnaires**. The participants completed the RST-PQ^6^ consisting of 65 items. This questionnaire measures three major systems: the BAS, 32 items; the BIS, 23 items related to anxiety; the FFFS, 10 ten items on active avoidance and fear. The BAS is a multidimensional system composed of the following facets: *Goal-Drive Persistence* (BAS-GDP, 7 items), *Reward Interest* (BAS-RI, 7 items), *Reward Reactivity* (BAS-RR, 10 items), and *Impulsivity* (BAS-I, 8 items). The total BAS (BAS-TOT) measure is obtained by summing the BAS-GDP, BAS-RI, BAS-RR, and BAS-I scores.

Participants respond on a scale from 1 (not at all) to 5 (highly) on how well each statement

describes them. Cronbach’s α values for BIS and FFFS were 0.88 and 0.79, respectively. Alpha values for the BAS-TOT, BAS-GDP, BAS-RI, BAS-RR, and BAS-I were, respectively, 0.87, 0.86, 0.79, 0.78, and 0.77.

We also administered the ECQ^7^, consisting of 27 questions tapping five sub traits of empathy. Participants respond to each item on a 4-point scale running from “definitely disagree” to “definitely agree.” In this questionnaire, the authors attempted to separate items into affective and cognitive empathy categories, in contrast with Baron-Cohen and Wheelwright^8^, claiming that empathy’s affective and cognitive components often co-occur and cannot be easily separated. The ECQ consists of five facets: (1) Cognitive Ability “CA” related to the skill or potential in the perspective-taking ; (2) Cognitive Drive “CD” related to the motivating interest in the perspective-taking ability; (3) Affective Ability “AA” related to the capacity or potential in recognizing, being sensitive to and sharing others’ emotional experiences; (4) Affective Drive “AD” related to the motivating interest in recognizing, being sensitive to and sharing others’ emotional experiences^9^; and (5) Affective Reactivity “AR” related to the ability in appropriate responding referring to another’s emotional experiences^8^. The Cronbach’s α values for the five facets ranged from 0.90 to 0.91 (see^7^). We found this questionnaire most appropriate for our purposes, seeing the valuable link between the cognitive aspect of empathy and embodiment/feeling ones^10^.

In the present study, from the above reported five measures, we derived the following principal scores: (*i*) *Cognitive Empathy* (CE) score, as the sum of Cognitive Ability and Cognitive Drive scores; (*j*) *Affective Empathy* (AE) score, as the sum of Affective Reactivity with Affective Ability and Affective Drive scores; (*k*) *Empathic Drive* (ED) score, as the sum of Cognitive Drive and Affective Drive scores; (*l*) *Total Empathic Ability* (TEA) score by summing the Cognitive Ability and Affective Ability scores, and (*m*) *Cumulative Total Empathy* (CTE) score by adding the Cognitive Empathy with Affective Empathy components. These five measures served for all statistical analyses of interest performed in this study. Participants also completed the state anxiety form of the State-Trait Anxiety Inventory (STAI-Y1)^11^.

**Section S2**

**Experimental tasks and treatments.** In a self-pain condition, participants were exposed to individually calibrated, short-lasting trains of painful and nonpainful electric stimuli delivered to the back of their right hand. In the other’s control, participants experienced empathy for the pain of the confederate seated next to which we delivered painful stimuli and nonpainful electric stimuli to the back of her right hand. A Digitimer DS5 Isolated Bipolar Constant Current Stimulator (Digitimer Clinical and Biomedical Research Instruments) generated electrical stimulations. We preferred electrical instead of thermal pain stimulations since the former responses have the advantage of faster onset and offset times than the latter. Since pain-specific evoked brain evoked responses involve the A-δ fibers and A-C terminals^12^, we used a planar concentric surface wasp point electrode (7 mm diameter). This electrode had a central platinum pin (WASP electrode, Specialty Developments, Germany), and we positioned it on the back of the participant’s right hand. We used concentric electrodes since conventional cutaneous bipolar electrodes usually recruit large-diameter sensory fibers. Concentric electrodes are reliable in producing pain-evoked potentials by stimulating superficial skin layer^13^. Additionally, Perchet et al.^14^ highlighted that the P2 component of the ERPs elicited by concentric electrodes was quite like that obtained with laser stimulations.

We used the e-prime 2.0 system to program the trial structure of the empathy for pain task. We benefited from a trial structure like that used by Rutgen et al.^15^, with a timeline adapted to make possible an HRV analysis of the EKG activity. A 19” color LCD monitor (1400 x 900 resolution and 75 Hz vertical refresh rate) presented visual stimuli, with the participants seated at a distance of 80 cm. Horizontal and vertical visual angles of all visual stimuli were 5.2° and 6.9°, with a mean luminance of 22.5 cd/m^2^. First, an arrow (1500 ms) was displayed indicating the upcoming electric stimulation, pointing either to the participant (arrow to the right) or to the confederate (arrow to the left side). An anticipatory colored spark visual cue (1000 ms) followed the arrow, indicating the intensity of the incoming electric stimulus, i.e., an orange spark for painful stimulation or a blue spark for non-pain stimulation. A dark screen lasting 3500 ms followed the visual spark cue. At the end of this interval, a colored cue spark (1000 ms) occurred with the onset of electric train pulse stimulation (duration from 18 to 30 ms): a red spark indicates a painful stimulus, a green spark a nonpainful one. After this stimulus, a dark screen for a fixed interval of 2500 ms occurred, followed by a fixation cross presented for 500 ms. Participants then rated their experienced pain (“how painful was this stimulus for you?”) on a numerical 7-point Likert scale (from “not at all” to “very painful”) to obtain a numerical pain score (NPS). Participants also rated the experienced unpleasantness (“How unpleasant was this stimulus for you?”) using a similar 7-point rating scale (ranging from “not at all” to “very unpleasant”) to obtain a numerical unpleasantness score (NUS). Equivalently, participants used a 7-point rating scale to infer the values for other pain (“How painful did it feel when the other person was stimulated?” They used the same scale to rate the inferred unpleasantness experienced by the confederate “How unpleasant did it feel when the other person was stimulated?”). Pain and unpleasantness ratings were presented in a quasi-random order. Only one-third of the painful trials were rated. The present trial structure enabled participants to know how and when the confederate was stimulated without direct observation of their actual reactions. In this way, we excluded influences resulting from direct observation, such as affective motor responses or emotional contagion.

In total, 36 trials were presented in random order for each condition: painful and nonpainful stimulation to the participant, painful and nonpainful stimulation to the confederate. To evaluate the level of relative pain and unpleasantness reduction induced by placebo treatment, we calculated numerical pain and unpleasantness difference scores (NPDSs and NUDSs) by subtracting NPSs, and NUSs rated during placebo from scores rated during pain. We used these difference scores for statistical analyses.

**Section S3**

**EEG Recordings and Wavelet Analysis.** EEG activity was recorded from 30 scalp sites (Fp1, Fp2, F7, F8, F3, F4, FT7, FT8, T3, T4, FC3, FC4, C3, C4, CP3, CP4, TP7, TP8, T5, T6, P3, P4, O1, O2, Fz, FCz, Cz, CPz, Pz, Oz; 10–20 system) using a 32-tin electrodes stretch Lycra cap (Electro-Caps, Eaton, OH, USA). We used linked earlobes [(A1+A2)/2] as a standard extracephalic reference with a ground electrode mounted between FPz and Fz. We employed the NuAmp acquisition system (Neuroscan Acquire 4.3, Compumedics Neuroscan Inc, Charlotte, North Carolina 28269, USA) with an online notch filter at 50 Hz. We kept electrode impedances < 5 kΩ, and we used standard tin electrodes to record horizontal and vertical EOGs from the right and left eye epicanthi and from supra- and infra-orbital positions of the right eye respectively. EEG was recorded in DC mode (sampling frequency = 1000 Hz) by using a gain of 200 (100 for eye channels) with a 0.01–150 Hz bandpass (Butterworth zero-phase filter with 24 dB/octave roll-off) and an online 50 Hz notch filter. We rejected the EEG activity above or below a threshold of 75 μV. In addition, two separate experimenters offline visually inspected the EEG recordings by using Brain Vision Analyzer 2.2.2. They did leave all the simultaneously recorded channels if they caught ocular, muscular, or movement artifacts on a single recording one^19,20^.

We removed horizontal and vertical EOGs and electromyographic (EMG) artifacts by extracting 1 to 3 out of 30 independent components (IC; using Infomax algorithm, Brain Products; Vision Analyzer 2.2.1, Gilching, Germany). We identified the EOGs and EMG (70-120 Hz)^21^ by visual (topographic) inspection of the independent component analysis (ICA) maps and comparisons with the EEG and EOG time series^22,23^. We discarded ICs without visible neural activity. After a visual re-examination, we identified EMG, eye movement, technical artifacts, artifact-free epoched data, and downsampled to 250 Hz. The EEG signals were then transformed to common average referencing to obtain reference-free recordings and identify small signal sources in relatively noisy recordings^24^.

We reconstructed the EEG trace into discrete, single-trial 1000 ms artifact-free epochs that were time-locked to the offset of painful electric train stimulus. The painful stimulus to the confederate was time-locked to the onset of a red-spark visual cue with a 500-ms prestimulus baseline.

For each of the four stimulation conditions (i.e., control and placebo, respectively for the participant and the confederate), we obtained from 33 to 36 artifact-free epochs. For each Treatment, we first calculated ERPs in self-pain and other pain conditions. The averaging epochs started 500 ms before stimulus onset and continued for 500 ms. We subtracted ERPs in each stimulus condition from the corresponding EEG epoch to remove the phase-locked EEG activity from the EEG data.

**Time-frequency (TF) transformation of the EEG responses.** We used a continuous wavelet transform to obtain a TF representation of the EEG signal. The wavelet transform differs from classical fixed window Fourier transform since the wavelet analysis adapted the width of its window as a function of frequency, and the convolution of the signal by a family of wavelets can provide a good TF resolution compromise of the signal^25,26^. It is essential to underline that the signal’s estimation of time and frequency is subjected to Heisenberg’s Uncertainty Principle. Estimates of low frequencies require wavelet transforms with wide windows, resulting in low-temporal and high-frequency resolutions. Vice-versa, estimates of high frequencies changes (for example, a transient variation in the signal) requires wavelet transforms using narrow windows, resulting in high-temporal and low-frequency resolutions.

In the present study, TF changes of the electrical activity elicited by the pain stimuli, single-trial data were convoluted with a complex Morlet wavelet (normalized to 1) defined as *w(t, f_0_) = (σ_t_*$\sqrt{\pi}$*)^-1/2^ e^(-t2/2σ^_t_^2^ ^)^ e^i2πf^_0_^t^,* where *t* is time, and *f* is frequency, which increased from 1 to 40 Hz with 40 frequency layers (1 Hz layer). The Morlet parameter *c=f_0_/σ_f_* (where *σ_f_ = 1/2πσ_t_*) was set to 5 (see^27,28^).

We estimated the square norm of the convolution of a complex wavelet w*(t, f_0_)* with the signal *s(t)*. We obtained these data with the following formula: *E(t, f_0_) =* |w*(t, f_0_) * s(t)*| *2*, where *E(t, f_0_) is the* time-varying energy of the signal in a frequency band around *f_0_* (wavelet duration 2σ_t_ of about 1.6 oscillatory periods at f_0_), with f_0_ ranging from 1 to 40 Hz in 1 Hz steps. We used a Gabor-normalized amplitude within a 1000 ms epoch in frequency steps of 1 Hz (logarithmic and linearly scaled for highlighting lower and higher frequencies). We considered the mean TF real power of the prestimulus period (between-500 and -50 ms) as a baseline level. For each frequency step, these baseline levels were subtracted from the prestimulus and poststimulus power. We obtained normalized non-phase-locked TF power representations separately for each pain treatment (i.e., control and placebo) of first-hand experience of pain and empathy for pain. We obtained TF of t-values for the following five EEG dominant sub frequencies and time-intervals: ϑ (4-8 Hz, 50-250 ms); α (9-13 Hz, 100-200 ms); β_1_ (14-21 Hz, 100-200 ms), β_2_ (22-32 Hz, 100-180 ms), γ (33-40 Hz, 120-180 ms). We then derived the frequencies corresponding to the maximum differences between pain and placebo treatments, i.e., 7, 12, 18, 31, and 39 Hz.

Consequently, we extracted the amplitude wavelet waveforms at each 7, 12, 18, 31, and 39 Hz frequency of interest to compute current source density (CSD) transforms^29,30^. We used this method to identify the primary topographic sources for each waveform interest, selecting the most sensitive recording leads. We calculated the CSD (μV/m²) by applying the spherical Laplace operator to the EEG voltage distribution on the surface of the head for each data point in time. This method entails the computation of the second spatial derivative of the EEG voltage assuming spherical head geometry. The CSD transform is reference-free, making CSD values independent from any previous choice of reference and invariant to any constant value added to the input EEG data. Thus, the application of the surface Laplacian considerably attenuates the effects of volume conduction on the EEG (spherical spline order m = 4, λ = 10^−5^, Legendre polynomial degree = 7). Since CSD mapping appears to be a valuable tool for spatially split smeared scalp-potential distribution due to simultaneously active generators, we used the CSD transform as a spatial filter to identify the source for each waveform interest^31^. Midline frontal (Fz), central Central (Cz), and parietal (Pz) sites were selected to reflect the topographical maximum of the amplitude at each frequency of interest. Thus, we used the mean of TF power calculated for each of the frequency bands mentioned above (i.e., ϑ, α, β_1_, β_2_, and γ) across midline recording sites for further statistical analyses. To reduce skew, averaged power values for each frequency of interest were submitted to a natural log transformation. These data served to calculate difference scores in the self-and other-related conditions by subtracting power scores obtained for Placebo treatment from the corresponding scores obtained for Control treatment.

**HR recordings.** An electrocardiogram (ECG) was obtained from two pure tin was recorded using two beryllium copper electrodes (1.5 cm in diameter) with a sample rate of 100 Hz. The electrodes were filled with electrolyte paste and attached to the sternum and the left rib cage. A ground plate electrode was attached to the right wrist. The ECG signal was detected using a Satem (modulab series 800) system that measured R–R intervals and yielded one data point every 10 ms. The R–R interval time series was first processed offline for artifact correction by using the smoothing tool of the Matlab R2014a. We excluded epochs considered too noisy or artifacts by gross muscular or respiratory movements. For each painful stimulation of Control and Placebo treatments delivered to the participant or the confederate, a 5 s epoch was extracted from the ongoing ECG, which included a prestimulus recording time of 1 s and a poststimulus recording of 4 s. In total, we selected a variable number of artifact-free epochs from 34 to 36, which corresponded to a total ECG recording time ranging from 2.8 to 3 min. We joined all recording epochs of the same Treatment into a single file temporal series for each participant. We performed the joining using an algorithm implemented on Matlab, making that time interval between the last R wave of the epoch n and the following R of the epoch n+1 had the mean inter-beat-interval (IBI) computed over all selected epochs for that Treatment. Finally, we processed the continuous ECG recording signal with Kubios HRV Analysis 3.0.2 software^32^ by applying the QRS detection algorithm included in this system.

We further inspected the ECG recording data to avoid artifacts caused by poor conductivity, large movements of the rib cage, and ventricular ectopic heartbeats. Less than 1.4% of beats were identified as artifacts, although we removed two cases for bad recordings and abnormal heartbeat activity. The Kubios HRV user’s guide manual describes HRV measures used in this study^33,34^. However, based on our previous findings^35^, we selected, among linear methods measures, time domain and frequency domain measures, and among nonlinear methods, the measure of sample entropy.

**Time- and frequency-domain HRV analyses.** Time-domain of R-R interval provided the mean IBI and standard deviation of normal-to-normal R-R interval (SDNN), which measures total variability in the series. Additionally, frequency domain analysis consisted of the spectral analysis of IBI. These analyses first required an IBI time series into an equidistantly sampled series by cubic spline interpolation with a sampling rate of 4 Hz. The IBI time series was divided into overlapping windows using Welsh’s periodogram to reduce leakage. Spectral analysis was then performed on these windowed segments using the Fast Fourier Transform (FFT). We obtained mean spectral power measures for the Low Frequency (LF, 0.04–0.15 Hz) and High Frequency (HF, 0.15–0.4 Hz) bands, respectively, indicating the influence of sympathetic and parasympathetic activity. We also obtained the LF/HF ratio as a balance measure of the autonomic nervous system (ANS). The guidelines of the Task Force of the European Society of Cardiology and The North American Society of Pacing and Electrophysiology^36^ served as a reference for the obtained measures. These data were normalized.

**Sample entropy.** Since classical linear HRV analysis alone provides few measures of the many factors involved in the regulation of the heart, among various nonlinear HRV measures, we choose sample entropy (SampEn) to capture extra information from IBI variability. The entropy of a system is defined as the amount of information produced by a random source of data representing the functioning system. SampEn is a modification of approximate entropy developed for estimating the complexity of physiological time-series signals^37^. SampEn, in comparison with approximate entropy, has the main advantage of data length independence. SampEn has been described as the negative natural logarithm for conditional properties that a series of data points a certain distance apart, *m*, would repeat itself at *m* + 1 where self-matches are not included in calculating the probability^38^. It is clear from its definition that SampEn will always be either zero or positive value. A smaller value of SaS a m p E n {\displaystyle SampEn} AmpEn indicates more [self-similarity](https://en.wikipedia.org/wiki/Self-similarity) in the data set or less noise. The SampEn is dependent on two parameters: the embedding dimension *m* and the tolerance *r*. In the current study, the generally used values for these parameters were: *m* = 2 and *r* = 0.2 SDNN^37,39^. The tolerance depends on SDNN to make this complexity measure insensitive to the overall variability level, making possible estimates of different subjects.

**Reduction of physiological variables.** In parallel with EEG difference scores, we also computed the following Control minus Placebo difference scores for the following HRV measures of interest: R-R mean intervals (∆R-R), the standard deviation of RR intervals (SDNN), low frequency (∆LF), high frequency (∆HF) power bands, ∆LF/∆HF) ratio, and sample entropy (∆SampEn). To reduce many autonomic and electrocortical indices, we performed five varimax-rotated Principal Components Analyses (PCAs, one for each of the five frequencies of interest), separately for Self-Pain and Other-Pain conditions, on the HR and EEG frequency indices. Each of the five PCA involved six HRV difference indices, as reported above, and three EEG difference scores as obtained at midline scalp sites of interest (Fz, Cz, and Pz). These analyses served to select (i) the EEG indices loading above the threshold of 0.40 in a factor together with HVR indices (j) to reduce problems of multicollinearity, for each EEG frequency of interest, in the subsequent analyses. Results of these preliminary analyses for Self-Pain and Other-Pain for ϑ, α, β_1_, β_2_, and γ EEG frequency bands of interest are reported in the main text. These analyses yielded three orthogonal factors with eigenvalues greater than 1, which were saved as standardized individual factor scores and used in the correlation analyses subsequently performed.

**Section S4**

**PCAs for Self-Pain Condition.** Common to all the separate PCA analyses of the self-pain condition was the first factor encompassing the frequency domain indices of LF power, HF power, and LH/HF ratio. This factor explained 32% of the variance for each separate PCA performed on HRV and each EEG frequency data. Due to the strong loadings on the frequency domain HR indices, we subsequently referred these factors to as “S_∆fHRV ϑ,” “S_∆fHRV α,” “S_∆fHRV β1”, “S_∆fHRV β2”, and “S_∆fHRV γ,” respectively for each frequency-of-interest analysis. We obtained each of these factors using the same HRV data together with each EEG-difference data of interest. Since each EEG-frequency data of interest yielded negligible loadings, we used only the first factor mentioned above and labeled it as “S_∆fHRV” in the subsequent analyses. We chose to avoid redundancy and collinearity problems in multiple correlation analyses.

The variance explained for each second factor was 19% for the ϑ, α, and γ analyses. For the ϑ analysis, the second factor included the negative loading of time-domain HRV and a high positive loading for the differences at midline (Fz, Cz, and Pz) power changes. We labeled this factor as “S_∆tHRV & ∆Midl-ϑPow,” indicating that Placebo treatment reduced the ϑ power and increased HR slowing. For the α analysis, the second factor encompassed a negative loading of the standard deviation of normal-to-normal (SDNN) R-R time interval, a positive loading of Sampling Entropy, and a negative loading of 12 Hz power at Cz changes. We mentioned this factor as “S_∆SD & ∆S-Entr & ∆Cz-αPow” a higher score indicated enhanced entropy and α power to Placebo treatment. Common to the β1 and β2 analyses were the loadings of the difference across midline sites. We referred them to as “S_∆Midl-β1Pow” and “S_∆Midl-β2Pow” and explained the 23% and 21% of the total variance. The PCA analysis, including γ power differences, yielded a negative loading for time-domain HRV and a positive loading for the differences at Cz and Pz. We mentioned this factor as “S_∆tHRV & ∆CzPz-γPow” (we multiplied it by -1 so that a higher score was more positive, indicating a reduced γ power and HR slowing to Placebo treatment).

The PCAs that included ϑ, α, β1, β2, and γ power differences had a third factor explaining the 16%, 15%, 17%, 16%, and 17% of the total variance. Each PCA analysis, including the ϑ, β1, and β2 power differences, yielded a similar third factor encompassing a positive loading of the standard deviation of R-R time interval (SDNN) and a negative loading of Sampling Entropy difference scores. Considering that each analysis used the same HRV data, we chose only the first of these factors for further correlation analyses. We labeled this factor as “S_∆SD & ∆S-Entr” (we multiplied it by -1 so that a higher score was more positive and indicated an increased HRV entropy). The PCA that included the α power differences yielded a third factor encompassing a negative loading for time-domain HRV and a positive loading for the EEG power differences at Cz and Pz. We labeled this factor as “S_∆tHRV & ∆CzPz-αPow” (it was multiplied by -1 so that a higher score indicated HR slowing and enhanced α power to Placebo treatment). The PCA that included the γ power differences highlighted a third factor that showed a negative loading of SDNN and a positive loading of Sampling Entropy difference scores, indicating that a higher score on this factor was positive. We referred to this factor as “S_∆SD & ∆S-Entr γ.”

**PCAs for Other-Pain.** The first factor was common to all PCAs for the other-pain condition that loaded all the HR frequency domain indices. This factor explained about 36% and 40% of the variance for EEG data that included the ϑ power and α scores, respectively. For each PCA, including β1, β2, and γ data analysis, the common HR frequency factor explained 37% of the variance (see the lower half of Tab. 1). This factor was multiplied by -1 so that a higher score on this factor indicates more vagal activity and more fractal-like dynamics. Given that loadings of frequency HRV indices were similar for each analysis, including the EEG frequency power of interest, we used only the first of these factors in the subsequent correlation analyses. We labeled this common factor as “O_∆fHRV.”

Each PCA analysis separately included the ϑ, β1, and β2 power differences and the same HRV difference indices, explaining the 17%, 18%, and 20% of the total variance. This factor, for each PCA, showed a postive loading of the upper-mentioned EEG-frequency power changes across midline sites and was labeled as “O_∆Midl-ϑPow,” “O_∆Midl-β1Pow,” “O_∆Midl-β2Pow.” A higher positive score on each factor indicated a reduced power to placebo treatment for the ϑ, β1, and β2 frequencies. The second factor was common to two separate EEG data sets, including α and the other including γ frequency power scores. Each explained, respectively, the 17% and 18% of the total variance and consisted of a positive loading of time HRV and Sampling Entropy. Higher scores of this factor indicated an increased HRV entropy and HR slowing to Placebo treatment. Of these factors, to avoid redundancy, only the first was used and referenced as “O_∆tHRV & ∆S-Entr. “

The third factor showed a positive loading of time HRV and sampling entropy common to each PCA conducted for ϑ, β1, and β2 data sets, which explained the 15%, 14%, and 14% of the total variance. We derived a second-factor loading of time HRV and sampling entropy from one dataset that included α, and the other included γ power differences. These two factors explained the 17% and 18% of the total variance. To avoid redundancy, only the first of these factors was used and referenced as “∆tHRV & ∆S-Entr“ (we did not multiply this factor by -1 because a higher score was already more positive). Higher positive scores of this common factor indicated a relatively reduced HRV entropy and HR increase to Placebo treatment. Smaller positive or higher negative scores indicated a relatively increased HRV entropy and HR slowing to placebo. For PCA, including HRV and α power data, the third factor explained the 15% of the total variance with positive loadings on α power difference scores at midline frontal (Fz) and central (Cz) regions. We labeled this factor as “O_∆FzCz-αPow.” For PCA that included HRV and γ power differences, the third factor had positive loadings on γ difference scores at midline (Fz, Cz, and Pz) sites and explained the 12% of the total variance. We referred to this factor as “O_∆Midl-γPow.” Higher scores of these last factors indicated a relatively reduced ϑ and γ powers to Placebo treatment.

**Section S5**

**Pain Ratings for Nonpainful Stimulation**

A repeated-measures ANOVA on pain rating scores to nonpainful stimulation for self-pain condition, with Gender as a between and Treatment as a within-subjects factor, displayed a significant main effect of Gender (Gender: *F*(1,60) = 6.37, p = 0.014, ɳ^2^_p_  = 0.096), indicating higher nonpainful stimulus sensitivity in women than men (see Table S5-1). No other effect was detected as significant (Treatment: *F*(1,60) = 0.001, p = 0.952, ɳ^2^_p_  = 0.0001; Gender x Treatment: *F*(1,60) = 1.16, p = 0.285, ɳ^2^_p_  = 0.019).

Similarly the ANOVA on pain rating scores to nonpainful stimulation for the other-pain condition did not show any significant effect (Gender: *F*(1,60) = 1.01, p = 0.319, ɳ^2^_p_  = 0.016; Treatment: *F*(1,60) = 0.07, p = 0.793, ɳ^2^_p_  = 0.001; Gender x Treatment: *F*(1,60) = 0.34, p = 0.564, ɳ^2^_p_  = 0.005).

Descriptive statistics of self-pain and other-pain rating scores for nonpainful stimulations in women and men participants are reported in Table S5-1.

**Section S6**

**Results**

**Table S6-1**

**Table S6-2**

**BEHAVIORAL INHIBITION SYSTEM (BIS**

**Table S6-3**

**Self-Pain Changes (S_NPDSs)**

**Self-Pain: Physiological factors predictors of BIS, pain, and unpleasantness reductions.** The three physiological factors significantly correlated with BIS (i.e., S_∆tHRV & ∆Midl-ϑPow, S_∆tHRV & ∆CzPz-αPow, and S_∆tHRV & ∆CzPz-γPow) were entered in a multiple regression model to find those that reliably predicted BIS scores by avoiding multicollinearity. We first calculate the correlation matrix among the three potential physiological predictors of BIS. In the correlation matrix, we observed correlations ranging between whopping values of 0.82 to 0.95, indicating multicollinearity. We detect the multicollinearity in our review of the parameter estimates results for Tol and VIF. We had three physiological factors with Tol values between 0.0012 and 0.0039 (i.e., well below the traditional 0.1 cutoff value) and VIF values between 220 and 850, far greater than the conventional 10 cutoffs. For completeness, we also reviewed the eigenvalue and condition index association. We observed that only the first factor (i.e., S_∆tHRV & ∆Midl-ϑPow) had an eigenvalue closer to 1, with the other two closer to zero, and the condition index of the third factor (i.e., S_∆tHRV & ∆CzPz-γPow) resulting of 66.35, i.e., above the traditional value of 30. We also noted that this third factor had a proportion of covariation above 0.98 with the other two factors. Thus, we excluded this third factor from further analyses (Table S5-1).

To combat the multicollinearity, we then tested the multiple regression model using the Elastic Nets method with Akaike’s information selection criterion^40,41^, using the predictors of BIS the two remaining factors (Table S5-2). This analysis yielded both factors of S_∆tHRV & ∆Midl-ϑPow and S_∆tHRV & ∆CzPz-αPow as potential predictors of BIS (F(2,59) = 9.01, p < 0.01, ɳ^2^_p_  = 0.190; R-Square = 0.234; Glmselect procedure, SAS-9.4^42^; Table S5-2).

Additionally, in the self-pain condition, only the three physiological factors correlated with S_NPDS (i.e., S_∆tHRV & ∆Midl-ϑPow, S_∆tHRV & ∆CzPz-αPow and S_∆tHRV & ∆CzPz-γPow). The S_∆tHRV & ∆CzPz-γPow factor had the main collinearity problem (see above). We excluded this factor in a multiple regression model testing predictors of pain reduction to PA treatment. Thus, we computed a multiple regression with two remaining physiological predictors of S_NPDS, with the Elastic Nets method for regularization and variable reductions (Table S5-3). This method yielded the S_∆tHRV & ∆Midl-ϑPow factor as the most reliable predictor of pain reduction (S_NPDS; (*F*(1,60) = 23.16, p < 0.001, ɳ^2^_p_  = 0.278; R-Square = 0.279). A similar method used for the selection, among two potential physiological predictors of unpleasantness reduction scores (S_NUDS) yielded again the S_∆tHRV & ∆Midl-ϑPow as the sole reliable predictor of S_NUDS (*F*(1,60) = 8.28, p < 0.01, ɳ^2^_p_  = 0.120; R-Square = 0.121).

**Table S6-4**

**TOTAL EMPATHY ABILITY (TEA)**

|  |  |  |  |  |  |  |  |
| --- | --- | --- | --- | --- | --- | --- | --- |
|  |  |  |  |  |  |  |  |
| **** |  |  |  |  |  |  |  |

**Table S5-5**

**OTHER-PAIN**

**Other-Pain: Physiological factors predictors of TEA and placebo pain reductions.** We want to detect physiological factors that reliably predicted the TEA trait in a multiple regression model. To do this, we first tested, using the same method reported above, the multicollinearity of the O_∆Midl-β2Pow and O_∆Midl-γPow factors, which we found significantly correlated with TEA scores. The correlation coefficient was 0.87 with Tol = 0.24, and VIF = 4.25. In addition, we observed that the O_∆Midl-β2Pow factor had an eigenvalue of about 1, whereas the O_∆Midl-γPow factor was close to 0.1 (Table S5-4). Thus, using as predictors the two physiological factors and as a criterion the TEA scores, we tested a multiple regression model with the Elastic Nets method by accounting for multicollinearity through Akaike’s information selection criterion^40,41^. This analysis selected only the O_∆Midl-β2Pow variable as the most reliable predictor of TEA (F(1,60) = 7.46, p < 0.01, ɳ^2^_p_  = 0.120; R-Square = 0.111; see Table S5-4).

We found that three physiological factors, namely O_∆f HRV, O_∆Midl-ϑPow, and O_∆Midl-β2Pow, were significantly correlated with O_NPDSs. We then tested collinearity in a multiple regression model using O_NPDS scores as a criterion and these three physiological factors as predictors. The correlation coefficients among these physiological factors ranged from -0.56 to 0.80. Tol values were not below the conventional cutoff of 0.1 (i.e., 0.30, 0.35, and 0.59) and VIF values were not above the conventional 10 cutoff (i.e, 1.69, 2.86, and 3.31). Since the Condition Indexes of each variable were not above the conventional cutoff value of 30 (i.e., 1.5, 2.2, and 3.6, respectively for each entered factor), we concluded that collinearity among these variables was not significant (Table S5-5). This conclusion was also validated by a multiple regression using the Elastic Nets method with Akaike’s information selection criterion that retained all the three physiological factors as reliable predictors of O_NPDSs (F(3,58) = 4.80, p < 0.01, ɳ^2^_p_  = 0.199; R-Square = 0.198).

**References**

1 Vecchio, A. & De Pascalis, V. ERP Indicators of Self-Pain and Other Pain Reductions due to Placebo Analgesia Responding: The Moderating Role of the Fight-Flight-Freeze System. *Brain Sciences* **11**, doi:<https://doi.org/> 10.3390/brainsci11091192 (2021).

2 Salmaso, D. & Longoni, A. M. Problems in the assessment of hand preference. *Cortex* **21**, 533-549, doi:10.1016/S0010-9452(58)80003-9 (1985).

3 Huang, Y. *et al.* Variations in resting frontal alpha asymmetry between high- and low-neuroticism females across the menstrual cycle. *Psychophysiology* **52**, 182-191, doi:<https://doi.org/10.1111/psyp.12301> (2015).

4 Gilbert, D. G., Dibb, W. D., Plath, L. C. & Hiyane, S. G. Effects of nicotine and caffeine, separately and in combination, on EEG topography, mood, heart rate, cortisol, and vigilance. *Psychophysiology* **37**, 583-595, doi:10.1111/1469-8986.3750583 (2000).

5 Cook, R. D. Detection of influential observation in linear regression. *Technometrics* **19**, 15-18, doi:10.1080/00401706.1977.10489493 (1977).

6 Corr, P. J. & Cooper, A. J. The Reinforcement Sensitivity Theory of Personality Questionnaire (RST-PQ): Development and Validation. *Psychological assessment* (2016).

7 Batchelder, L., Brosnan, M. & Ashwin, C. The Development and Validation of the Empathy Components Questionnaire (ECQ). *PLOS ONE* **12**, e0169185, doi:10.1371/journal.pone.0169185 (2017).

8 Baron-Cohen, S. & Wheelwright, S. The Empathy Quotient: An Investigation of Adults with Asperger Syndrome or High Functioning Autism, and Normal Sex Differences. *Journal of Autism and Developmental Disorders* **34**, 163-175, doi:10.1023/B:JADD.0000022607.19833.00 (2004).

9 Decety, J. Dissecting the Neural Mechanisms Mediating Empathy. *Emotion Review* **3**, 92-108, doi:10.1177/1754073910374662 (2011).

10 Zahavi, D. Empathy, Embodiment and Interpersonal Understanding: From Lipps to Schutz. *Inquiry* **53**, 285-306, doi:10.1080/00201741003784663 (2010).

11 Spielberger, C. D., Gorsuch, R., Lushene, R., Vagg, P. R. & Jacobs, G. A. *Manual for the statetrait anxiety inventory (form Y)*. (Consulting Psychologist Press, 1988).

12 Cruccu, G. *et al.* Conduction velocity of the human spinothalamic tract as assessed by laser evoked potentials. *NeuroReport* **11** (2000).

13 Katsarava, Z. a. *et al.* A Novel Method of Eliciting Pain-Related Potentials by Transcutaneous Electrical Stimulation. *Headache: The Journal of Head and Face Pain* **46**, 1511-1517, doi:<https://doi.org/10.1111/j.1526-4610.2006.00446.x> (2006).

14 Perchet, C. *et al.* Do we activate specifically somatosensory thin fibres with the concentric planar electrode? A scalp and intracranial EEG study. *PAIN* **153**, 1244-1252, doi:<https://doi.org/10.1016/j.pain.2012.03.004> (2012).

15 Rütgen, M., Seidel, E. M., Riečanský, I. & Lamm, C. Reduction of Empathy for Pain by Placebo Analgesia Suggests Functional Equivalence of Empathy and First-Hand Emotion Experience. *The Journal of Neuroscience* **35**, 8938, doi:10.1523/JNEUROSCI.3936-14.2015 (2015).

16 Donchin, E. Surprise!… Surprise? *Psychophysiology* **18**, 493-513, doi:<https://doi.org/10.1111/j.1469-8986.1981.tb01815.x> (1981).

17 Kirsch, I. & Weixel, L. J. Double-blind versus deceptive administration of a placebo. *Behav Neurosci* **102**, 319-323 (1988).

18 Price, D. D. *et al.* An analysis of factors that contribute to the magnitude of placebo analgesia in an experimental paradigm. *Pain* **83**, 147-156 (1999).

19 Hagemann, D., Naumann, E. & Thayer, J. F. The quest for the EEG reference revisited: a glance from brain asymmetry research. *Psychophysiology* **38**, 847-857 (2001).

20 Barlow, J. S. in *Handbook of electroencephalography and clinical neurophysiology, Revised series. Clinical applications of computer analysis of EEG and other neurophysiological signals* Vol. 2 (eds F.H. Lopes da Silva, W. Storm van Leeuwen, & A. Rémond) 15-62 (Elsevier, 1986).

21 Cacioppo, J. T. *Principles of psychophysiology: Physical, social, and inferential elements*. (Cambridge University Press, 1990).

22 Delorme, A., Sejnowski, T. & Makeig, S. Enhanced detection of artifacts in EEG data using higher-order statistics and independent component analysis. *Neuroimage* **34**, 1443-1449, doi:10.1016/j.neuroimage.2006.11.004 (2007).

23 Olbrich, S., Jödicke, J., Sander, C., Himmerich, H. & Hegerl, U. ICA-based muscle artefact correction of EEG data: What is muscle and what is brain?: Comment on McMenamin et al. *Neuroimage* **54**, 1-3, doi:10.1016/j.neuroimage.2010.04.256 (2011).

24 Ludwig, K. A. *et al.* Using a common average reference to improve cortical neuron recordings from microelectrode arrays. *Journal of neurophysiology* **101**, 1679-1689, doi:10.1152/jn.90989.2008 (2009).

25 Mouraux, A., Guerit, J. & Plaghki, L. Non-phase locked electroencephalogram (EEG) responses to CO 2 laser skin stimulations may reflect central interactions between A∂-and C-fibre afferent volleys. *Clinical neurophysiology* **114**, 710-722 (2003).

26 Mouraux, A. & Iannetti, G. D. Across-trial averaging of event-related EEG responses and beyond. *Magnetic Resonance Imaging* **26**, 1041-1054, doi:<https://doi.org/10.1016/j.mri.2008.01.011> (2008).

27 Grossmann, A., Kronland-Martinet, R. & Morlet, J. in *Wavelets. Inverse problems and theoretical imaging* (eds J. M. Combes, A. Grossmann, & P. Tchamitchian) 2-20 (Springer, 1990).

28 Tallon-Baudry, C., Bertrand, O., Delpuech, C. & Pernier, J. Oscillatory γ-Band (30–70 Hz) Activity Induced by a Visual Search Task in Humans. *The Journal of Neuroscience* **17**, 722, doi:10.1523/JNEUROSCI.17-02-00722.1997 (1997).

29 Tenke, C. E. & Kayser, J. Generator localization by current source density (CSD): Implications of volume conduction and field closure at intracranial and scalp resolutions. *Clinical Neurophysiology* **123**, 2328-2345, doi:<https://doi.org/10.1016/j.clinph.2012.06.005> (2012).

30 Kayser, J. & Tenke, C. E. On the benefits of using surface Laplacian (current source density) methodology in electrophysiology. *Int J Psychophysiol* **97**, 171-173, doi:10.1016/j.ijpsycho.2015.06.001 (2015).

31 McFarland, D. J., McCane, L. M., David, S. V. & Wolpaw, J. R. Spatial filter selection for EEG-based communication. *Electroencephalography and Clinical Neurophysiology* **103**, 386-394, doi:<https://doi.org/10.1016/S0013-4694(97)00022-2> (1997).

32 Tarvainen, M. P., Niskanen, J.-P., Lipponen, J. A., Ranta-Aho, P. O. & Karjalainen, P. A. Kubios HRV–heart rate variability analysis software. *Computer methods and programs in biomedicine* **113**, 210-220, doi:10.1016/j.cmpb.2013.07.024 (2014).

33 Tarvainen, M. P. & Niskanen, J.-P. in *Biosignal Analysis and Medical Imaging Group (BSAMIG)* Vol. 109 (2006).

34 Young, H. & Benton, D. We should be using nonlinear indices when relating heart-rate dynamics to cognition and mood. *Scientific reports* **5**, 16619, doi:10.1038/srep16619 (2015).

35 De Pascalis, V. & Scacchia, P. The influence of reward sensitivity, heart rate dynamics and EEG-delta activity on placebo analgesia. *Behavioural Brain Research* **359**, 320-332, doi:<https://doi.org/10.1016/j.bbr.2018.11.014> (2019).

36 Malik, M. *et al.* Heart rate variability: Standards of measurement, physiological interpretation, and clinical use. *European Heart Journal* **17**, 354-381, doi:10.1093/oxfordjournals.eurheartj.a014868 (1996).

37 Richman, J. S. & Moorman, J. R. Physiological time-series analysis using approximate entropy and sample entropy. *American Journal of Physiology-Heart and Circulatory Physiology* **278**, H2039-H2049, doi:10.1152/ajpheart.2000.278.6.H2039 (2000).

38 Yentes, J. M. *et al.* The appropriate use of approximate entropy and sample entropy with short data sets. *Annals of biomedical engineering* **41**, 349-365 (2013).

39 Fusheng, Y., Bo, H. & Qingyu, T. Approximate entropy and its application to biosignal analysis. *Nonlinear Biomedical Signal Processing: Dynamic Analysis and Modeling, Volume 2*, 72-91, doi:10.1109/9780470545379.ch3 (2001).

40 Mallows, C. L. Some comments on C p. *Technometrics* **15**, 661-675 (1973).

41 Hocking, R. R. A Biometrics invited paper. The analysis and selection of variables in linear regression. *Biometrics* **32**, 1-49 (1976).

42 Schreiber-Gregory, D. N. Ridge Regression and multicollinearity: An in-depth review. *Model Assisted Statistics and Applications* **13**, 359-365, doi:10.3233/MAS-180446 (2018).

**Data availability**

The data supporting the findings of this study are available from the corresponding author upon reasonable request.
